# Construction and Analysis of lncRNA-Mediated ceRNA Network in Nasopharyngeal Carcinoma Based on Weighted Correlation Network Analysis

**DOI:** 10.1155/2020/1468980

**Published:** 2020-10-10

**Authors:** Zhenning Zou, Shuguang Liu, Yanping Ha, Bowan Huang

**Affiliations:** ^1^Department of Pathology, Guangdong Medical University, Zhanjiang, China; ^2^Department of Pathology, The Eighth Affiliated Hospital of Sun Yat-sen University, Shenzhen, China; ^3^Department of Anesthesiology, Zhanjiang Central Hospital, Guangdong Medical University, Zhanjiang, China

## Abstract

Increasing evidence indicated that aberrant expression of long noncoding RNAs (lncRNAs) are involved in tumorigenesis of nasopharyngeal carcinoma (NPC). The purpose of this study was to construct a lncRNA-mediated ceRNA network based on weighted correlation network analysis (WGCNA). First, modules with highly correlated genes were identified from GSE102349 via WGCNA, and the preservation of the modules was evaluated by GSE68799. Then, the differentially expressed lncRNAs and mRNAs identified from GSE12452 which belonged to the same WGCNA modules and the differentially expressed miRNAs identified from GSE32960 were used to construct a ceRNA network. The prognostic value of the network was evaluated by survival analysis. Furthermore, a risk score model for predicting progression-free survival (PFS) of NPC patients was established via LASSO-penalized Cox regression, and the differences in the expression of the lncRNAs between high- and low-risk groups were investigated. Finally, 14 stable modules were identified, and a ceRNA network composed of 11 lncRNAs, 15 miRNAs, and 40 mRNAs was established. The lncRNAs and mRNAs in the network belonged to the turquoise and salmon modules. Survival analysis indicated that ZNF667-AS1, LDHA, LMNB2, TPI1, UNG, and hsa-miR-142-3p were significantly correlated with the prognosis of NPC. Gene set enrichment analysis indicated that the upregulation of ZNF667-AS1 was associated with some immune-related pathways. Besides, a risk score model consisting of 12 genes was constructed and showed a good performance in predicting PFS for NPC patients. Among the 11 lncRNAs in the ceRNA network, SNHG16, SNHG17, and THAP9-AS1 were upregulated in the high-risk group of NPC, while ZNF667-AS1 was downregulated in the high-risk group of NPC. These results will promote our understanding of the crosstalk among lncRNAs, miRNAs, and mRNAs in the tumorigenesis and progression of NPC.

## 1. Introduction

Nasopharyngeal carcinoma (NPC) is a fatal malignancy arising from the nasopharynx epithelium. It is characterized by a distinctive geographical and ethnic distribution with a high incidence of NPC in Southern China, Southeast Asia, and Middle East/North Africa [1]. Southern China accounts for 71% of new cases worldwide [2]. It was estimated that there were 129,079 new cases and 72,987 deaths around the world in 2018 [3]. With the emergence of advanced diagnostic methods and improvement of treatment methods, especially the application of intensity-modulated radiotherapy, the prognosis of NPC has been dramatically improved over the past decades [2]. Nevertheless, a portion of patients still had adverse outcomes because of locoregional recurrence and distant metastasis [4, 5]. Hence, it is necessary to explore the molecular mechanisms underlying NPC and develop more effective therapeutic approaches to reduce the occurrence of posttherapy complications and improve the prognosis of patients with NPC.

LncRNAs are referred to transcripts longer than 200 nucleotides. Although lacking protein-coding ability, lncRNAs can interact with RNA or DNA molecules via base pairing, even with double-stranded DNA, and form networks with DNA, protein complexes, and RNA [6]. LncRNAs serve as competitive endogenous RNAs (ceRNAs) and implicated in the occurrence and progression of a variety of tumors, including NPC. For example, lncRNA H19 was suggested to regulate EZH2 expression by interacting with miR-630 and promotes cell invasion in NPC [7]. LncRNA HOXC13-AS was reported to positively affect cell proliferation and invasion in NPC via modulating miR-383-3p/HMGA2 axis [8]. LncRNA FAM225A was revealed to promote NPC tumorigenesis and metastasis by sponging mir-590-3p/mir-1275 and upregulate ITGB3 [9]. LncRNA SNHG1 was shown to antagonize the effect of miR-145a-5p on the downregulation of NUAK1 in NPC [10]. The ceRNA hypothesis supposed that any RNA transcript that harbors the miRNA recognition elements (MREs) can sequester miRNAs from other targets sharing the same MREs, thereby regulating the expression of target genes [11, 12]. However, there are still few comprehensive studies concerning lncRNA-mediated ceRNA network in NPC based on high-throughput data.

Weighted correlation network analysis (WGCNA) is a widely used and efficient system biology method [13]. This method can identify clusters (modules) of highly correlated genes based on high-dimensional data and can further identify hub genes closely related to external phenotypic traits, which may be potential diagnostic and therapeutic markers. This process is different from many other bioinformatics methods because it does not rely on priori defined gene sets or pathways [14]. WGCNA has been applied in various biological processes, such as cancer, genetics, and brain imaging data analysis [15]. As for NPC, four miRNAs, including hsa-miR-142-3p, hsa-miR-150, hsa-miR-29b, and hsa-miR-29c, were obtained as prognostic markers by combining univariate Cox regression analysis with WGCNA [16]. Ge et al. identified several gene modules with different biological functions related to NPC through WGCNA analysis [17]. However, to our knowledge, no study has previously constructed a lncRNA-mediated ceRNA network based on WGCNA in NPC.

In the present study, we obtained RNA-Seq data of GSE102349 and performed WGCNA to enrich modules associated with NPC. Subsequently, we used mRNA profiling data (GSE12452) and miRNA profiling data (GSE32960) to identify differentially expressed genes (DEGs) and differentially expressed miRNAs (DEmiRNAs). Through annotation of DEGs and intersection with WGCNA modules, we obtained differentially expressed lncRNAs (DElncRNAs) and differentially expressed mRNAs (DEmRNAs) in the WGCNA modules. Then, those DElncRNAs, DEmiRNAs, and DEmRNAs were used to construct a lncRNA-miRNA-mRNA ceRNA network based on LncBase v.2 [18] and miRTarBase [19] databases. Next, survival analysis was performed to explore the prognostic characteristics of the ceRNA network. Gene Ontology (GO) and Kyoto Encyclopedia of Genes and Genomes (KEGG) pathways enrichment analyses were implemented for the WGCNA modules, lncRNAs, and mRNAs in the ceRNA network. Gene set enrichment analysis (GSEA) was also carried out for the prognostic lncRNAs in the ceRNA network. Moreover, we constructed a LASSO-penalized Cox regression model for progression-free survival (PFS) prediction. The differences in the expression of the lncRNAs and mRNAs in the ceRNA network between high- and low-risk groups were investigated. The results of this study will promote our understanding of the crosstalk among lncRNAs, miRNAs, and mRNAs in the tumorigenesis and progression of NPC.

## 2. Materials and Methods

### 2.1. Data Collection

Four public datasets of NPC (GSE102349, GSE68799, GSE32960, and GSE12452) were obtained from the Gene Expression Omnibus database (GEO) (http://www.ncbi.nlm.nih.gov/geo/). GSE102349, performed on the RNA-Seq platform of GPL11154 (Illumina HiSeq 2000), including 113 NPC tissues, was used to conduct WGCNA. GSE68799, performed on the same platform as GSE102349, consisting of 42 NPC and 4 non-NPC tissues, was used for module preservation analysis [20]. GSE32960, performed on the miRNA profiling platform of GPL14722 (CapitalBio, Inc., Beijing, China), containing 312 paraffin-embedded NPC and 18 paraffin-embedded normal nasopharyngeal tissues, was used to identify differentially expressed miRNAs (DEmiRNAs) between NPC and normal tissues. GSE12452 performed on the mRNA profiling platform of GPL570 [HG-U133_Plus_2] (Thermo Fisher Scientific, Inc.), comprising 31 NPC and 10 normal nasopharyngeal tissues, was used to identify differentially expressed genes (DEGs) between NPC and normal tissues. Since these data are downloaded from the public database, the consent of the ethics committee is not required.

### 2.2. Weighted Correlation Network Analysis

WGCNA was performed based on GSE102349 with “WGCNA” package in the R software (version 3.5.0; http://www.r-project.org) for a weighted correlation network construction. The gene expression levels were measured by log2-transformed fragments per kilobase million (FPKM). Only genes with the highest 25% variance of expression values among samples were included in WGCNA. The outliers were identified and removed by hierarchical clustering analysis. Proper soft thresholding power was obtained based on the criterion of approximate scale-free topology. Next, “one-step network construction and module detection” function was used to construct a signed coexpression network and identify modules associated with NPC. The preservation of the modules was examined by GSE68799. The preservation value “medianRank” and “Zsummary.pres” (Z-score) were calculated using the *nPermutations* of 200. In general, if Z-score is lower than 2, the module will be regarded as no evidence of preservation, while Z − score > 10 will be regarded as high preservation. Furthermore, the associations between each module and the clinical traits were analyzed by Pearson's correlation test. *P* < 0.05 was considered significant.

### 2.3. Differential Expression Analysis between NPC and Normal Tissues

To identify DEmiRNAs and DEGs between NPC and normal tissues, we performed differential expression analysis based on GSE32960 and GSE12452 using “limma” package [21]. Before analysis, miRNA expression values were log2-transformed and quantiled normalized across multiple arrays. Gene expression values of GSE12452 were calculated by the method described in our previous study [22]. The DEmiRNAs and DEGs with false discovery rate (FDR) < 0.05 and ∣log fold change (FC) | >0.5 were considered significant and used for subsequent analysis.

### 2.4. Identification of lncRNAs and Construction of the ceRNA Network

The lncRNAs and mRNAs in the WGCNA modules were defined and annotated based on the Gencode lncRNA annotation file (gencode.v31.long_noncoding_RNAs.gtf.gz) from the GENCODE database (https://www.gencode-genes.org). Following, a lncRNA-mediated ceRNA network was constructed based on the hypothesis that lncRNAs can interact with target miRNAs and invoked miRNA sponges to regulate the activity of mRNAs [11]. According to the hypothesis, both lncRNAs and mRNAs had a negative correlation with the corresponding miRNAs. We speculated that the mRNAs may be the target genes of lncRNAs that belong to the same WGCNA module, so we first obtained up- and downregulated DElncRNAs and DEmRNAs in NPC by taking the intersection of the DEGs and the genes from the WGCNA modules. Then, we used the validated lncRNA-miRNA interaction data from LncBase v.2 experimental module [18] to predict target miRNAs for DElncRNAs. The target miRNAs were matched with DEmiRNAs to acquire up- and downregulated miRNAs for the ceRNA network. Subsequently, we used the experimentally verified miRNA-mRNA interaction data from miRTarBase database [19] to predict target mRNAs for those up- and downregulated miRNAs. After that, the target mRNAs were matched with the DEmRNAs in the WGCNA modules to obtain mRNAs for the ceRNA network. Thus, a ceRNA network based on the WGCNA modules was constructed and visualized by the Cytoscape v3.6 software. The workflow of building the ceRNA network was provided in Figure S1.

### 2.5. Survival Analysis for the lncRNAs, miRNAs, and mRNAs in the ceRNA Network

There were 88 NPC samples with PFS data in GSE102349. PFS was defined as the time from the date of diagnosis to the date of objective tumor progression or death from any cause [23]. There were 312 NPC samples with prognostic information in the dataset GSE32960. The prognostic information included overall survival (OS), disease-free survival (DFS), and distant metastasis-free survival (DMFS). To explore the relationship between the expression level of lncRNAs, miRNAs, and mRNAs in the ceRNA network and survival time, the samples were divided into two groups of high expression and low expression according to the median value. Then, the Kaplan-Meier survival curve combined with a log-rank test was performed to compare the survival difference between the high- and low-expression groups. Survival analysis was implemented using “survival” and “survminer” packages [24, 25]. *P* < 0.05 was considered to be statistically significant.

### 2.6. Functional and Pathway Enrichment Analysis for the Genes in the WGCNA Modules and ceRNA Network

To explore the potential biological functions for the genes in the WGCNA modules and lncRNA-mediated ceRNA network, the genes were submitted to GO and KEGG pathway enrichment analysis using “clusterProfiler” package [26]. For GO analysis, the cut-off criteria were *P* < 0.05 and *q* < 0.05. For KEGG analysis, the cut-off criterion was *P* < 0.05.

### 2.7. GSEA for the Prognostic lncRNAs in the ceRNA Network

To explore the potential pathways of the prognostic lncRNAs in the ceRNA network, the samples in the GSE102349 and GSE12452 were categorized into two groups of high expression and low expression according to the median value of the prognostic lncRNAs. Then, GSEA was performed by “clusterprofiler” package using the annotated gene set, “c2.cp.kegg.v7.0.symbols.gmt” from the Molecular Signature Database (MSigDB, http://software.broadinstitute.org/gsea/msigdb/index.jsp) as the reference gene set. False discovery rate (FDR) < 0.05 was chosen as the threshold.

### 2.8. Construction of Risk Score Model

To construct a risk score model for PFS prediction, the DEGs with false discovery rate (FDR) < 0.05 and ∣log fold change (FC) | >1 in the high preserved WGCNA modules were subjected to LASSO-penalized Cox regression analysis by “glmnet” package [27, 28] in R. The function “cv.glmnet” was used to compute 10-fold cross-validation for the Cox model. “Lambda.min” was selected as the optimal *λ* value. The prognostic genes and their coefficients were generated to calculate the risk score (RS) for each sample. The RS formula was constructed as follows: risk score = ∑(*β* × expr). Here, *β* represents the coefficient of a gene, and expr represents the expression level of the gene. According to the median value of the RS, 88 samples in GSE102349 were classified into high- and low-risk groups. Then, the Kaplan-Meier survival curve combined with a log-rank test was performed to evaluate the survival differences between high- and low-risk groups using “survival” and “survminer” packages. The receiver operating characteristic (ROC) curve, the area under the curve (AUC), and the C-index were applied to assess the prognostic accuracy of the risk score model using “survivalROC” package [29]. Besides, the distribution of the risk score, survival status, and expression patterns of the prognostic gene signatures were explored in GSE102349. After that, the prognostic genes were subjected to univariate Cox analysis and multivariate Cox analysis. *P* < 0.05 was considered statistically significant. Using “forestplot” package [30], a forest plot was used to illustrate the results of hazard ratio (HR), 95% CI (confidence interval), and corresponding *P* value. Finally, we compared the expression levels of the lncRNAs in the ceRNA network between high- and low-risk groups in GSE102349 using two-sided Student's *t*-test. *P* < 0.05 was considered to be statistically significant.

## 3. Results

### 3.1. Weighted Correlation Network Analysis

**Figure 1:**
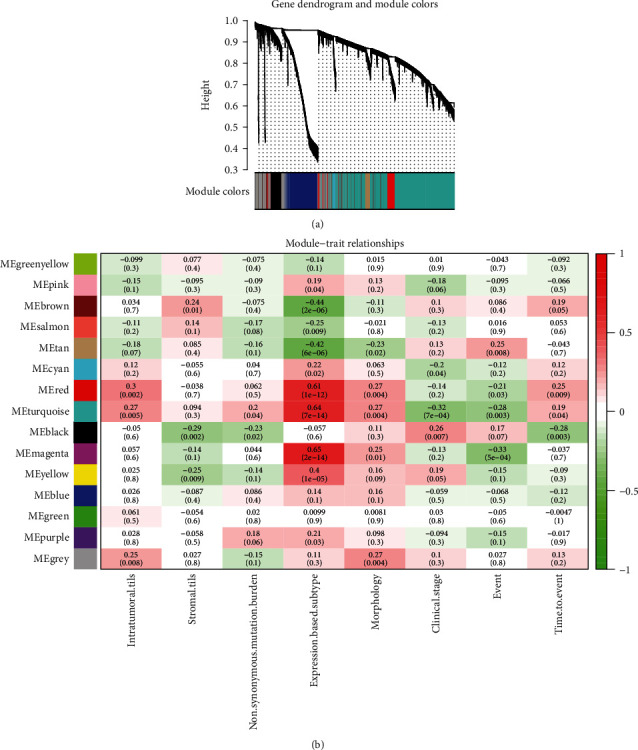
Weighted gene coexpression network analysis. (a) Hierarchical clustering dendrogram of the 14 modules. The branches of the tree represent the clusters of genes. Colors below the tree display the gene modules that correspond to the clusters. (b) Heatmap of the correlation between module eigengenes and clinical traits of NPC. Each row corresponds to a module eigengene, column to a trait. Each cell contains the corresponding correlation and *P* value. NPC: nasopharyngeal carcinoma.

A weighted correlation network of NPC based on GSE102349 was constructed using “WGCNA” package. After removing three samples (GSM2735294, GSM2735309, and GSM2735313) which were considered abnormal after sample clustering analysis, 110 samples were retained for further analysis (Fig. S2). We chose the soft-thresholding power of *β* as three, which was the lowest power for which the scale-free topology fit index (scale-free *R*^2^) achieved 0.9 (Fig. S3). Then, a weighted correlation network with 14 modules was constructed. The sizes of the modules ranged from 52 (cyan module) to 2045 (turquoise module) genes. A hierarchical clustering dendrogram (tree) was generated with the color assignment (Figure 1(a)). The genes labeled with “gray” represented background genes that were not assigned to any coexpressive gene modules. Following, the preservation of the modules were examined using GSE68799. Results showed that the 14 modules were stable because their Z-scores were all higher than two (Fig. S4). Among them, ten modules (blue, turquoise, yellow, brown, red, cyan, magenta, black, tan, and purple) were high preserved (Z − score > 10). Besides, we calculated the correlations between the 14 modules and the clinical traits (Figure 1(b)). The results showed that three modules (cyan, turquoise, and black) were significantly correlated with clinical stage, four modules (tan, red, turquoise, and magenta) were significantly correlated with the event, and four modules (brown, red, turquoise, and black) were significantly correlated with time to event (*P* < 0.05).

### 3.2. Identification of DEmiRNAs and DEGs in NPC Tissues

**Figure 2:**
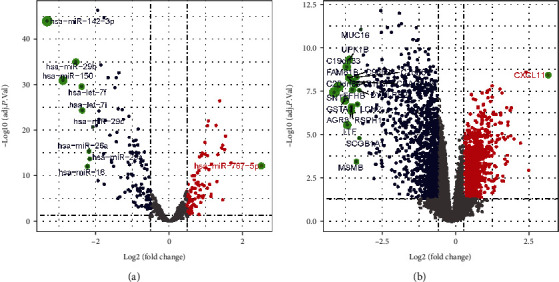
Volcano plots of (a) DEmiRNAs and (b) DEGs. Spots on the right represent upregulated genes, and spots on the left represent downregulated genes. False discovery rate (FDR) < 0.05 and ∣log fold change (FC) | >0.5 were considered significant. DEmiRNAs: differentially expressed miRNAs; DEGs: differentially expressed genes.

Based on the miRNA profiling dataset GSE32960, we identified a total of 196 DEmiRNAs, including 87 (44.4%) upregulated miRNAs and 109 (55.6%) downregulated miRNAs. Meanwhile, based on the mRNA profiling dataset GSE12452, we identified 2371 DEGs, including 1191 (50.2%) upregulated genes and 1180 (49.8%) downregulated genes. The DEmiRNAs (Figure 2(a)) and DEGs (Figure 2(b)) were depicted in volcano plots, respectively.

### 3.3. Identification of lncRNAs and Construction of the ceRNA Network

**Figure 3:**
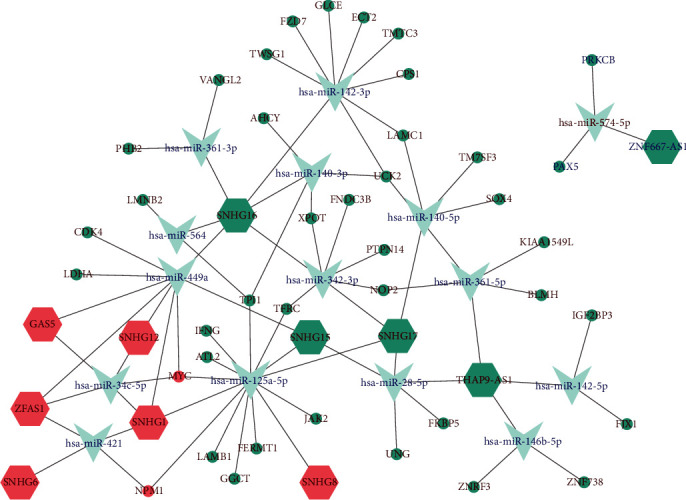
LncRNA-mediated ceRNA network of NPC. Hexagon, V shaped, ellipse denote lncRNA, miRNA, and mRNA, respectively. The nodes labeled with red represent upregulated, and the nodes labeled with blue represent downregulated. The color of the node indicates the WGCNA module it belongs to. NPC: nasopharyngeal carcinoma.

Using GENCODE database, we identified 65 lncRNAs in the WGCNA modules. However, after matching with the DEGs from GSE12452, LncBase v.2, and miRTarBase databases, only 11 lncRNAs were retained to construct the lncRNA-mediated ceRNA network (Table 1). Of them, ZNF667-AS1 was downregulated in NPC, and the others (GAS5, SNHG1, SNHG12, SNHG15, SNHG16, SNHG17, SNHG6, SNHG8, THAP9-AS1, and ZFAS1) were upregulated in NPC. As demonstrated in Figure 3, we eventually constructed a ceRNA network composed of 11 lncRNAs (Table 1), 15 miRNAs (Table S1), and 40 mRNAs (Table S2) from turquoise and salmon modules.

### 3.4. Survival Analysis for the lncRNAs, miRNAs, and mRNAs in the ceRNA Network

**Figure 4:**
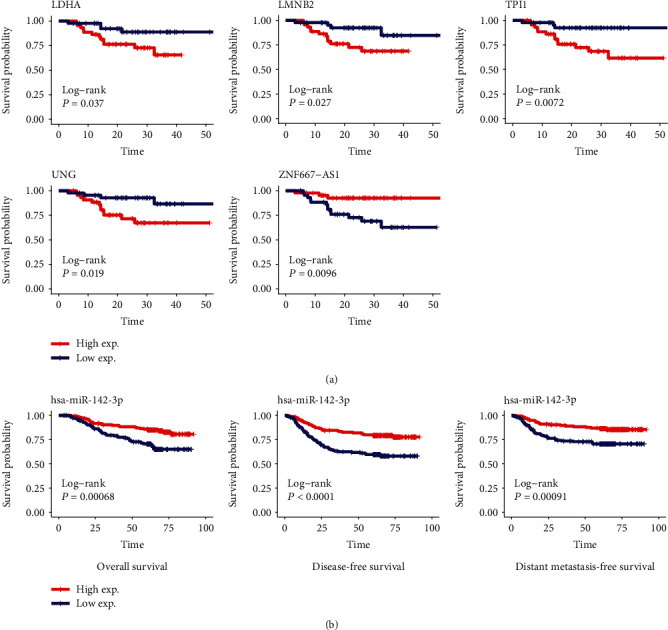
Survival analysis for the lncRNAs, miRNAs, and mRNAs in the ceRNA network. LDHA, LMNB2, TPI1 and UNG, and ZNF667-AS1 were significantly correlated with PFS of patients with NPC (a). Hsa-miR-142-3p were significantly correlated with OS, DFS, and DMFS of patients with NPC (b). NPC: nasopharyngeal carcinoma; PFS: progression-free survival; OS: overall survival; DFS: disease-free survival; DMFS: distant metastasis-free survival.

Kaplan-Meier survival analysis was performed to explore the prognostic value of the lncRNAs, miRNAs, and mRNAs in the ceRNA network. Results showed that four mRNAs, including LDHA, LMNB2, TPI1, and UNG, were negatively correlated with PFS, and lncRNA ZNF667-AS1 was positively correlated with PFS. Patients in the high-expression group of ZNF667-AS1 had longer PFS than those in the low-expression group (Figure 4(a)). Additionally, hsa-miR-142-3p was found to be positively correlated with OS, DFS, and DMFS (Figure 4(b)).

### 3.5. Functional and Pathway Enrichment Analysis for the Genes in the WGCNA Modules

The potential biological function of the genes in the WGCNA modules was investigated by GO and KEGG pathway enrichment analysis. Results indicated that the genes in the modules were associated with different functional categories and pathways (Fig. S5A-B). For example, “Wnt signaling pathway” was enriched in the black module; “Small cell lung cancer” was enriched in the brown module; “p53 signaling pathway” was enriched in the brown and tan modules; “PI3K-Akt signaling pathway” was enriched in the brown, turquoise, and yellow modules; “Epstein−Barr virus infection” was enriched in the cyan and turquoise modules; “IL−17 signaling pathway” was enriched in the green modules; “Amoebiasis” was enriched in the purple, turquoise, and yellow modules; “Cell cycle,” “Oocyte meiosis,” and “DNA replication” were enriched in the tan module; “Protein digestion and absorption” was enriched in the yellow module.

### 3.6. Functional and Pathway Enrichment Analysis for the Genes in the ceRNA Network

**Figure 5:**
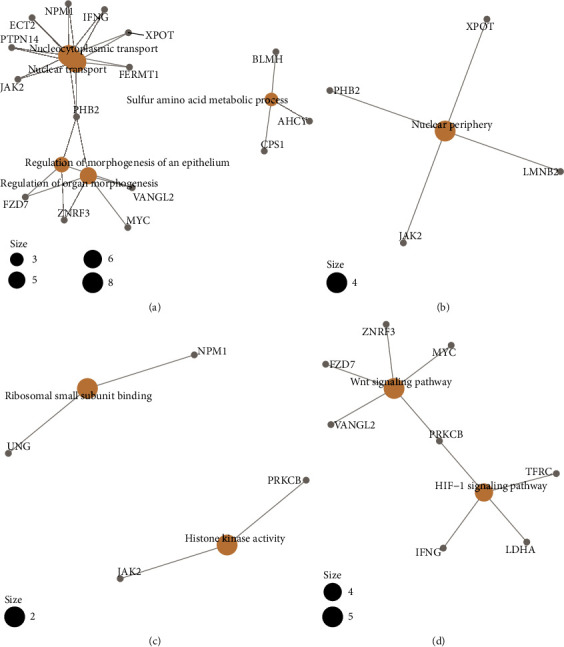
Network of representative GO terms and KEGG pathways. GO term enrichment analysis for biological process (a), molecular function (b), cellular component (c). KEGG pathway analysis (d). GO: gene ontology; KEGG: Kyoto Encyclopedia of Genes and Genomes.

Through GO and KEGG pathway enrichment analysis, we also studied the potential biological functions of the genes in the ceRNA network. Results showed that the genes were significantly enriched in 76 GO terms for biological processes (BP), two GO terms for molecular function (MF), and one GO term for cellular components (CC) (Table S3). For BP, enriched terms included “nucleocytoplasmic transport,” “nuclear transport,” “regulation of organ morphogenesis,” “regulation of morphogenesis of an epithelium,” and “sulfur amino acid metabolic process”. For CC, the enriched term was “nuclear periphery.” For MF, the enriched terms included “ribosomal small subunit binding” and “histone kinase activity.” Additionally, KEGG pathway enrichment analysis indicated that “Wnt signaling pathway” and “HIF-1 signaling pathway” were significantly enriched. We displayed the results as 4 networks that depict the representative GO terms and KEGG pathways (Figure 5).

### 3.7. GSEA for ZNF667-AS1

**Figure 6:**
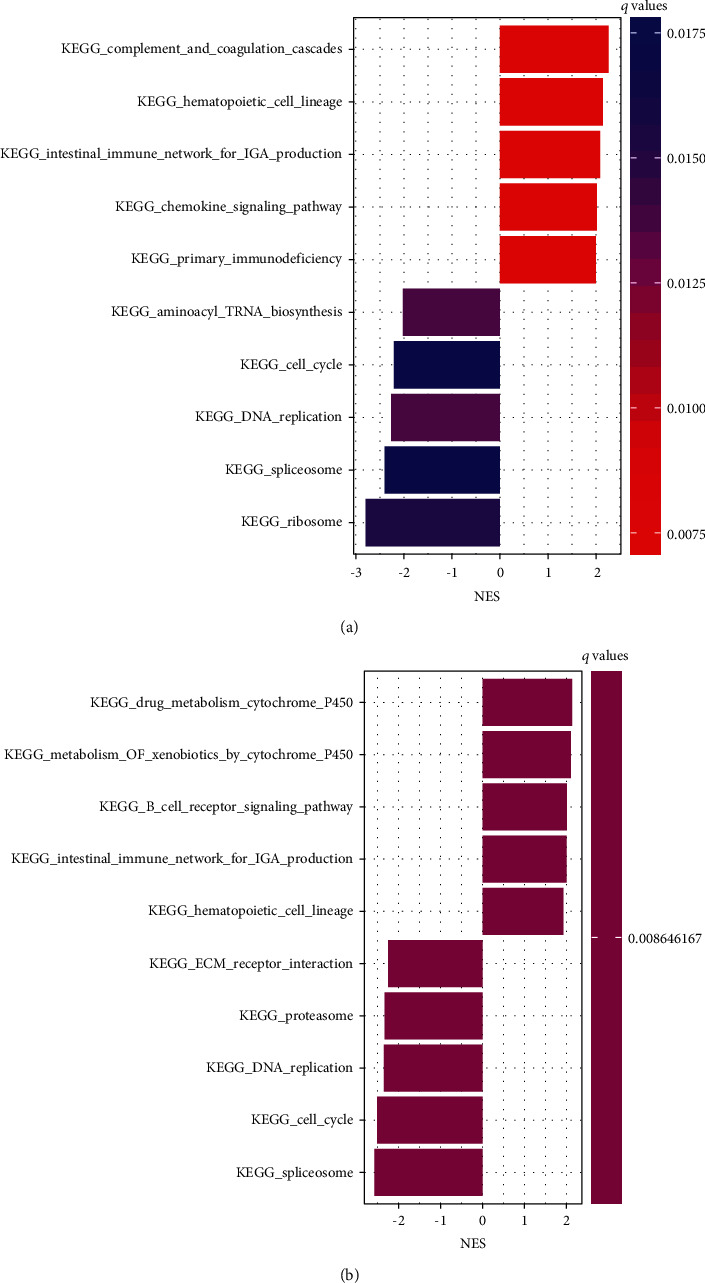
Gene set enrichment analysis (GSEA) for ZNF667-AS1 using GSE102349 (a) and GSE12452 (b). The pathway with normalized enrichment score (NES) > 0 may be activated when ZNF667-AS1 was upregulated; otherwise, it may be suppressed.

As shown in the above analysis, of the 11 lncRNAs, only ZNF667-AS1 was correlated with PFS significantly. To better understand the role of ZNF667-AS1 in NPC, GSEA was performed on GSE102349 and GSE12452. The results showed that 58 pathways were enriched in GSE102349 (Table S4), and 54 pathways were enriched in GSE12452 (Table S5). Further analysis revealed that these two datasets shared 44 pathways.  Figure 6 showed the top five activated pathways and five suppressed pathways when ZNF667-AS1 was upregulated and downregulated. The upregulation of ZNF667-AS1 was associated with “intestinal immune network for IgA production,” “hematopoietic_cell_lineage,” “B cell receptor signaling pathway,” “T cell receptor signaling pathway,” “leukocyte transendothelial migration,” and other pathways. The downregulation of ZNF667-AS1 was related to “DNA replication,” “cell cycle,” “P53 signaling pathway,” “spliceosome,” and other pathways.

### 3.8. Construction of Risk Score Model

**Figure 7:**
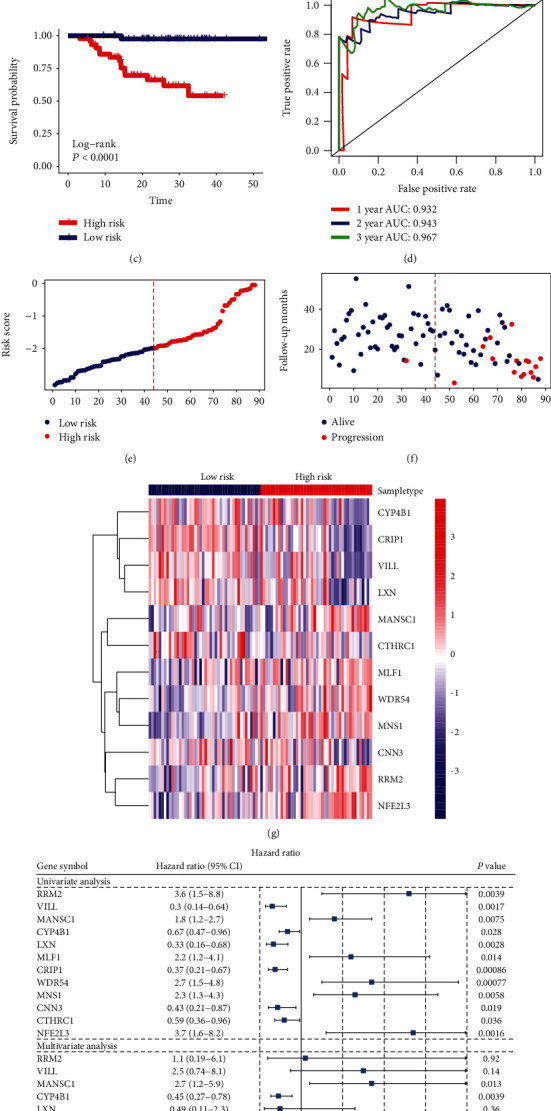
Construction of risk score model based on LASSO-penalized Cox regression analysis. LASSO coefficients at varying levels of penalty were shown in (a). Plot of the cross-validation error rates was shown in (b). The left vertical line in the plot shows the CV-error curve hits its minimum. The right vertical line shows the most regularized model with CV-error within 1 standard deviation of the minimum. Base on the LASSO-penalized Cox regression of PFS, the RS for each sample was calculated. Then, according to the median value of RS, the samples were separated into high- and low-risk groups. Kaplan-Meier survival analysis for the risk score model was shown in (c). Time-dependent ROC curve analysis for the risk score model was shown in (d). Risk score distribution of samples was shown in (e). Survival profile of samples was shown in (f). A heatmap was generated to show the expression of 12 prognostic genes in NPC (g). A forrest plot of the univariate and multivariate Cox regression analysis for the prognostic genes associated with PFS was shown in (h). NPC: nasopharyngeal carcinoma; CI: confidence interval; PFS: progression-free survival.

**Figure 8:**
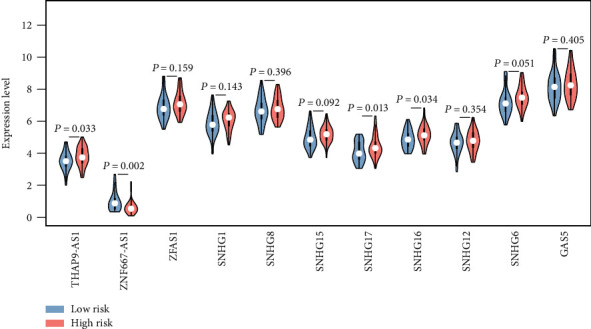
Comparison of the expression levels of the lncRNAs in the ceRNA network between high- and low-risk groups in NPC. NPC: nasopharyngeal carcinoma.

LASSO-penalized Cox regression analysis along with 10-fold cross-validation was conducted to build risk score model for PFS prediction (Figures 7(a) and 7(b)), which resulted in the identification of 12 prognostic genes: RRM2, VILL, MANSC1, CYP4B1, LXN, MLF1, CRIP1, WDR54, MNS1, CNN3, CTHRC1, and NFE2L3. Risk score for each sample was calculated based on the following formula: RS = 0.07785695 × expr_RRM2_ − 0.05729219 × expr_VILL_ + 0.15392701 × expr_MANSC1_ − 0.02185837 × expr_CYP4B1_ − 0.05553768 × expr_LXN_ + 0.19470422 × expr_MLF1_ − 0.44565573 × expr_CRIP1_ + 0.22242843 × expr_WDR54_ + 0.39891041 × expr_MNS1_ − 0.27436890 × expr_CNN3_ − 0.03554977 × expr_CTHRC1_ + 0.08434817 × expr_NFE2L3_. According to the median value of RSs, 88 samples in the dataset GSE102349 were separated into high- and low-risk groups. Kaplan-Meier analysis showed that patients in the high-risk group had poorer PFS than those in the low-risk group (Figure 7(c)). Time-dependent ROC analysis at varying follow-up times was used to explore the prognostic capacity of the risk score model for PFS. Results showed that the AUC received 0.932, 0.943, and 0.967 at 12, 24, and 36 months (Figure 7(d)). The C-index for the model was 0.891. Therefore, this model has good PFS prediction ability in GSE102349. The distribution of the risk score indicated that patients in the high-risk group had a worse outcome of PFS than those in the low-risk group (Figures 7(e)–7(f)), and the former tended to have overexpression of RRM2, MANSC1, MLF1, WDR54, MNS1, and NFE2L3, whereas the latter tended to have overexpression of VILL, CYP4B1, LXN, CRIP1, CNN3, and CTHRC1 (Figure 7(g)). Besides, multivariate Cox regression analysis indicated that MANSC1, CYP4B1, MLF1, CRIP1, MNS1, CNN3, and CTHRC1 were independent prognostic factors associated with the DFS in NPC (Figure 7(h)). Among the 11 lncRNAs in the ceRNA network, SNHG16, SNHG17, and THAP9-AS1 were upregulated in the high-risk group of NPC, while ZNF667-AS1 was downregulated in the high-risk group of NPC (Figure 8).

## 4. Discussion

LncRNAs, function as ceRNAs by binding miRNAs, have been reported to be involved in the physiological and pathological processes of various diseases. One of the most effective methods to predict the function of ceRNAs was to construct a ceRNA network based on the high-throughput data together with bioinformatic tools and computational approaches [31]. However, the comprehensive analysis of lncRNA-mediated ceRNA regulatory network in NPC remains scarce.

In the present study, we conducted WGCNA using RNA-Seq data of GSE102349 to enrich modules associated with NPC. Finally, we built a weighted correlation network composed of 14 modules. Through enrichment analysis, we found that the modules were related to some terms and pathways that had been previously reported by us or other researchers, such as “Wnt signaling pathway,” “Small cell lung cancer,” “PI3K−Akt signaling pathway,” “Epstein−Barr virus infection,”, “Cell cycle,” and “DNA replication” [17, 22, 32].

To better understand the potential roles of lncRNAs in NPC, we identified lncRNAs from the WGCNA modules. Combining with mRNA expression profile data, we identified the up- and downregulated lncRNAs and mRNAs in the same module. The mRNAs in the same module may be the regulatory targets for lncRNAs, so we used the lncRNAs and mRNAs in the same module to construct a ceRNA network. To improve the reliability of the ceRNA network, we identified the DEmiRNAs from GSE32960. Following, LncBase v.2 database was utilized to predict target miRNAs for lncRNAs, and miRTarBase was utilized to predict target mRNAs for miRNAs. Finally, we developed a ceRNA network composed of 11 lncRNAs, 15 miRNAs, and 40 mRNAs from turquoise and salmon modules. It is noteworthy that the genes in the ceRNA network were significantly enriched in the “Wnt signaling pathway” and “HIF-1 signaling pathway” which had been shown to be related to the development of NPC. For instance, Wang et al. [33] revealed that Wnt/*β*-catenin signaling (including *β*-catenin, cyclin D1, c-Myc, and MMP-7) and p-eIF4E expression were elevated in NPC compared with noncancerous nasopharyngeal epithelial tissues and associated with clinical characteristics of NPC patients. Sung et al. [34] showed that HIF-1 alpha, HIF-2 alpha, CA IX, and VEGF were frequently coexpressed in NPC biopsies and associated with poor outcomes after radiotherapy.

Among the 40 mRNAs in the ceRNAs, four mRNAs were found to have a significant impact on the prognosis of NPC, including LDHA, LMNB2, TPI1, and UNG. High expression of LDHA, LMNB2, TPI1, and UNG indicated unfavorable outcomes in patients with NPC. LDHA is upregulated in NPC tissues and cells, and it was reported to be an independent adverse prognostic factor of NPC [35, 36]. Through inhibition of LDHA, miR-34b-3 and miR-449a suppressed NPC progression and metastasis [37]. Therefore, the ceRNA network was reliable because the regulatory relationship between LDHA and miR-449a was indicated in our ceRNA network. LMNB2, a B type nuclear lamin, binds to the C-terminus of MCM7 and competes with the binding of the tumor suppressor RB protein, thus regulates human non-small-cell lung cancer progression [38]. TPI1, an enzyme that catalyzes the interconversion of DHAP and G3P in glycolysis and gluconeogenesis, might be a novel prognostic factor to evaluate gastric cancer patients' survival [39, 40]. UNG is a critical mediator of pemetrexed sensitivity in lung cancer [41]. The role and mechanism of LMNB2, TPI1, and UNG in NPC remain elusive.

Among the 15 miRNAs in the ceRNAs, hsa-miR-142-3p was demonstrated to be downregulated in NPC, and a high level of hsa-miR-142-3p indicated favorable prognosis, which was consistent with the previous studies [16, 42]. Li Y et al. revealed that miR-142-3p was epigenetically silenced by EZH2-recruited DNMT1 and suppress NPC cell metastasis and EMT through targeting ZEB2 [43]. However, Qi et al. reported that miR-142-3p inhibits the expression of SOCS6 and promotes cell proliferation in NPC [44]. Therefore, the role of miR-142-3p in the progression of NPC deserves further studied.

Among the 11 lncRNAs in the ceRNAs, ZNF667-AS1 was found to be related to the PFS of patients with NPC. ZNF667-AS1, also known as MORT, is located in 19q13.43. Dysregulated expression of ZNF667-AS1 has been reported in many tumors, including breast cancer, cervical cancer, laryngeal squamous cell carcinoma, and esophageal squamous cell carcinoma [45–48]. Li et al. found that ZNF667-AS1 reduces tumor invasion and metastasis in cervical cancer by counteracting microRNA-93-3p-dependent PEG3 downregulation [46]. Dong et al. demonstrated that aberrant hypermethylation-mediated downregulation of ZNF667-AS1 and ZNF667 correlates with progression and prognosis of esophageal squamous cell carcinoma [47]. Further, ZNF667-AS1 was revealed to be silenced by aberrant DNA methylation in 22 of 33 of TCGA cancer types [49]. In this study, ZNF667-AS1 was shown to be downregulated in NPC compared with normal tissues, and it was downregulated in the high-risk patients with poor prognosis. The expression level of ZNF667-AS1 was positively correlated with PFS of NPC. Combined with the above literature, we speculated that the downregulation of ZNF667-AS1 might also be caused by methylation in NPC, but it needs further molecular experiments to prove this hypothesis. Besides, we found that ZNF667-AS1 may regulate the expression of PRKCB and PAX5 through competitively binding to hsa-miR-574-5p. What is more, GSEA analysis showed that ZNF667-AS1 was associated with some important pathways related to tumorigenesis. Therefore, ZNF667-AS1 might act as a tumor suppressor and participate in the occurrence and development of NPC. To the best of our knowledge, the present study was the first to demonstrate the relation between ZNF667-AS1 and NPC using bioinformatics analysis.

In the present study, we constructed a risk score model for predicting PFS of NPC patients. This model consists of 12 genes RRM2, VILL, MANSC1, CYP4B1, LXN, MLF1, CRIP1, WDR54, MNS1, CNN3, CTHRC1, and NFE2L3. Among them, RRM2, MANSC1, MLF1, WDR54, MNS1, and NFE2L3 were associated with high risk of poor prognosis, while VILL, CYP4B1, LXN, CRIP1, CNN3, and CTHRC1 were associated with low risk of poor prognosis. Multivariate Cox regression analysis indicated that MANSC1, CYP4B1, MLF1, CRIP1, MNS1, CNN3, and CTHRC1 were independent prognostic factors associated with the DFS in NPC. Kaplan-Meier survival analysis, ROC curve and the C-index demonstrated that the predictive capability of the risk model was successful in GSE102349. Among the prognostic genes in the model, RRM2 encodes a subunit of ribonucleotide reductase, which catalyzes the conversion of ribonucleotides into deoxyribonucleotides. Overexpression of RRM2 predicts an unfavorable prognosis for patients with NPC [50]. CYP4B1 encodes a member of the cytochrome P450 superfamily of enzymes, which catalyze many reactions involved in drug metabolism and synthesis of cholesterol, steroids, and other lipids. Although CYP4B1 was detected in NPC tissues [51], the role of CYP4B1 is little known. In addition to RRM2 and CYP4B1, the relationship between NPC and other prognostic genes in the risk model has not been reported in the literature.

What is more, we analyzed the expression differences of lncRNAs in the ceRNA network between two risk groups. Results showed that SNHG16, SNHG17, and THAP9-AS1 were upregulated in the high-risk group of NPC, while ZNF667-AS1 was downregulated in the high-risk group of NPC. SNHG16 was regarded as an oncogene and associated with neuroblastoma, bladder cancer, colorectal cancer, esophageal squamous cell carcinoma, and hepatocellular carcinoma [52–54]. Zhang et al. suggested that SNHG16 promotes tumor progression through acting as an endogenous “sponge” by competing with miR-140-5p, thereby regulating target ZEB1 [53]. SNHG17 was reported to promote gastric cancer progression by epigenetically silencing of p15 and p57, and it was an unfavorable prognostic factor in colorectal cancer and gastric cancer [55, 56]. Extensive overexpression of THAP9-AS1 was observed in pancreatic ductal adenocarcinoma which is associated with poor clinical outcomes [57]. Function as ceRNA, THAP9-AS1 facilitated YAP expression by sequestrating miR-484, and it can bind YAP to inhibit phosphorylation-mediated inactivation by LATS1 [57]. The function and mechanism of those identified lncRNAs in NPC warrant further study.

Altogether, based on the WGCNA method and ceRNA hypothesis, combined with bioinformatics database, this study constructed a lncRNA-mediated ceRNA network, which is of great significance for us to identify lncRNAs with important biological significance in NPC, especially the lncRNAs' relevance to prognosis and risk, and to understand the regulatory mechanism and function of lncRNAs. The lncRNAs, miRNAs, and mRNAs identified in this study may serve as candidate diagnostic and therapeutic targets for NPC and provide clues for further in-depth research in the future.

It should be acknowledged that there were certain limitations to the present study. Firstly, only 11 differential expressed lncRNAs were obtained from the integrated analysis of RNA-Seq data and mRNA profiling data. Some lncRNAs may be ignored, for only a portion of the possible lncRNAs was included in HG-U133 Plus 2.0 platform. Secondly, the sample size of GSE102349 was relatively limited. There were only 113 NPC samples included in GSE102349, and only 88 samples had survival information; thus, research with large sample sizes, high-quality high-throughput data are required to verify our findings in the future. Thirdly, due to the lack of important clinical information such as gender and age, we cannot confirm the independence of the risk model. Moreover, due to the absence of similar NPC public datasets with survival information, external validation was not conducted. Finally, although we have constructed a ceRNA network using multiple datasets and experimental-based databases, the regulatory relationship among lncRNAs, miRNAs, and mRNAs still requires to verify using molecular experiments in vivo and in vitro.

## 5. Conclusions

Collectively, we constructed a ceRNA network based on WGCNA. The network can reflect the mutual regulated relationships among lncRNAs, miRNAs, and mRNAs, and it can reflect the changes in their expression level between NPC and normal tissues. This ceRNA network provided novel insights into the understanding of the crosstalk among lncRNAs, miRNAs, and mRNAs in the tumorigenesis and progression of NPC. Moreover, we developed a LASSO-penalized Cox regression model for NPC that may contribute to predict PFS of NPC patients. Furthermore, we identified several potential prognostic biomarkers and high-risk biomarkers from the ceRNA network. Further evaluation of those biomarkers is needed by well-designed scientific experiments in the future.

## Figures and Tables

**Table 1:** The differential expressed lncRNAs in the ceRNA network.

Module color	Symbol	LogFC	adj.*P*.Val	Group	Target miRNA
Salmon	ZFAS1	0.804750464	2.10*E*-04	Up	hsa-miR-34c-5p; hsa-miR-421; hsa-miR-449a
Salmon	SNHG12	0.710805371	0.007409834	Up	hsa-miR-34c-5p; hsa-miR-449a
Salmon	GAS5	0.902974269	1.73*E*-05	Up	hsa-miR-34c-5p; hsa-miR-449a
Salmon	SNHG6	0.615722821	0.003071714	Up	hsa-miR-421
Salmon	SNHG1	1.067332781	2.98*E*-07	Up	hsa-miR-125a-5p; hsa-miR-34c-5p; hsa-miR-421; hsa-miR-449a
Salmon	SNHG8	1.065333272	1.10*E*-05	Up	hsa-miR-125a-5p
Turquoise	SNHG16	0.722248011	9.24*E*-04	Up	hsa-miR-140-3p; hsa-miR-142-3p; hsa-miR-342-3p; hsa-miR-361-3p; hsa-miR-449a; hsa-miR-564
Turquoise	SNHG17	0.627392375	0.001876364	Up	hsa-miR-125a-5p; hsa-miR-140-5p; hsa-miR-28-5p; hsa-miR-342-3p
Turquoise	SNHG15	0.668944147	1.22*E*-04	Up	hsa-miR-125a-5p; hsa-miR-28-5p; hsa-miR-449a
Turquoise	THAP9-AS1	0.759104166	0.003243755	Up	hsa-miR-142-5p; hsa-miR-146b-5p; hsa-miR-28-5p; hsa-miR-361-5p
Turquoise	ZNF667-AS1	-1.117518496	5.97*E*-07	Down	hsa-miR-574-5p

Abbreviation: LogFC: log_2_-fold change; adj.*P*.Val: *P* value adjusted for multiple testing.

## Data Availability

The data used for analysis in this study are available from the Gene Expression Omnibus database.

## Supplementary Materials

Supplementary Materials Figure S1: workflow of the construction of lncRNA-mediated ceRNA network. Figure S2: sample clustering analysis to detect outliers. Figure S3: network topology analysis for various soft-thresholding powers (*β*). The scale-free fit index (left) and the mean connectivity (right) were displayed as a function of the soft-thresholding power. The red line represents the standard line when *R*^2^ reached a value of 0.9. Figure S4: the medianRank and Zsummary statistics of module preservation of GSE102349 modules in GSE68799 modules (*y*-axis) vs. module size (*x*-axis). Figure S5: enriched GO terms and KEGG pathways for WGCNA modules. (A) GO term enrichment for biological processes, molecular functions, and cellular components. (B) KEGG pathway analysis. The number on the *x*-axis is the number of enriched genes. The circle size indicates the ratio of genes in each term; colors correspond to Padj. *P* values were adjusted by the Benjamini-Hochberg method. GO, Gene Ontology; KEGG, Kyoto Encyclopedia of Genes and Genomes; Padj, adjusted *P* value. Table S1: DEmiRNAs in the ceRNA network. Table S2: DEmRNAs in the ceRNA network. Table S3: enriched GO terms and KEGG pathways for the genes in the ceRNA network. Table S4: gene set enrichment analysis (GSEA) for ZNF667-AS1 using GSE102349. Table S5: gene set enrichment analysis (GSEA) for ZNF667-AS1 using GSE12452.
