# Supplementary material for: Construction and Analysis of lncRNA-Mediated ceRNA Network in Nasopharyngeal Carcinoma Based on Weighted Correlation Network Analysis
Source: Biomed Res Int. 2020 Oct 10;2020:1468980. doi: 10.1155/2020/1468980 (PMC7569441; doi:10.1155/2020/1468980)
Supplement: Supplementary Materials — Figure S1: workflow of the construction of lncRNA-mediated ceRNA network. Figure S2: sample clustering analysis to detect outliers. Figure S3: network topology analysis for various soft-thresholding powers (β). The scale-free fit index (left) and the mean connectivity (right) were displayed as a function of the soft-thresholding power. The red line represents the standard line when R2 reached a value of 0.9. Figure S4: the medianRank and Zsummary statistics of module preservation of GSE102349 modules in GSE68799 modules (y-axis) vs. module size (x-axis). Figure S5: enriched GO terms and KEGG pathways for WGCNA modules. (A) GO term enrichment for biological processes, molecular functions, and cellular components. (B) KEGG pathway analysis. The number on the x-axis is the number of enriched genes. The circle size indicates the ratio of genes in each term; colors correspond to Padj. P values were adjusted by the Benjamini-Hochberg method. GO, Gene Ontology; KEGG, Kyoto Encyclopedia of Genes and Genomes; Padj, adjusted P value. Table S1: DEmiRNAs in the ceRNA network. Table S2: DEmRNAs in the ceRNA network. Table S3: enriched GO terms and KEGG pathways for the genes in the ceRNA network. Table S4: gene set enrichment analysis (GSEA) for ZNF667-AS1 using GSE102349. Table S5: gene set enrichment analysis (GSEA) for ZNF667-AS1 using GSE12452. [file 1468980.f1.pdf]

Figure S1

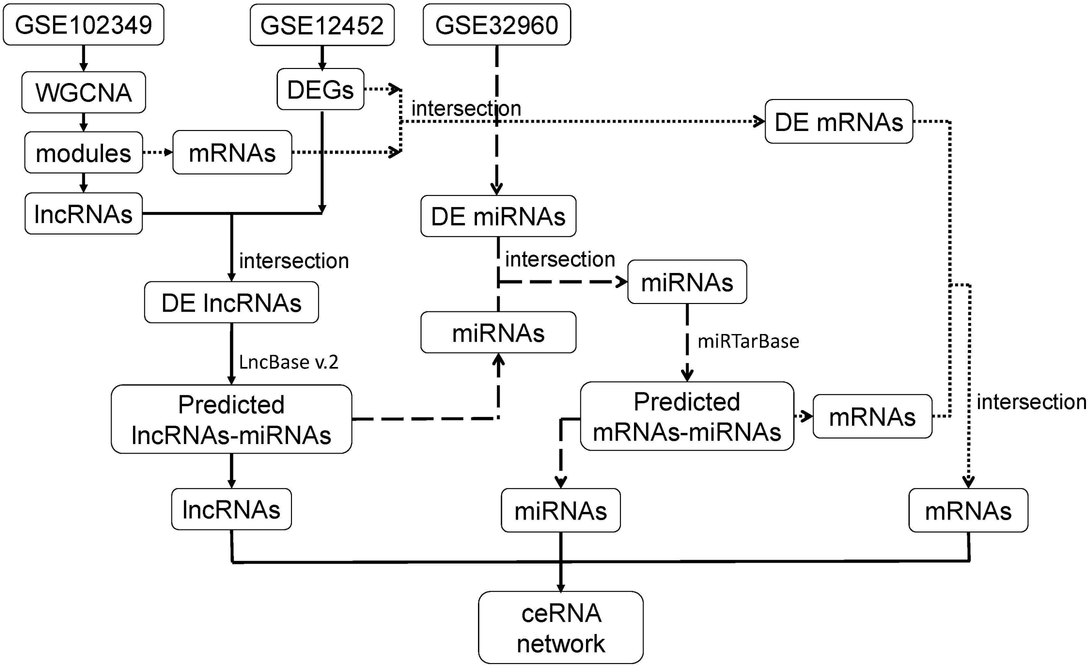
GSE102349
GSE12452
GSE32960
WGCNA
DEGs
intersection
DE mRNAs
modules
mRNAs
lncRNAs
DE miRNAs
intersection
intersection
miRNAs
DE lncRNAs
miRNAs
miRTarBase
LncBase v.2
Predicted lncRNAs-miRNAs
Predicted mRNAs-miRNAs
mRNAs
intersection
lncRNAs
miRNAs
mRNAs
ceRNA network

Figure S2

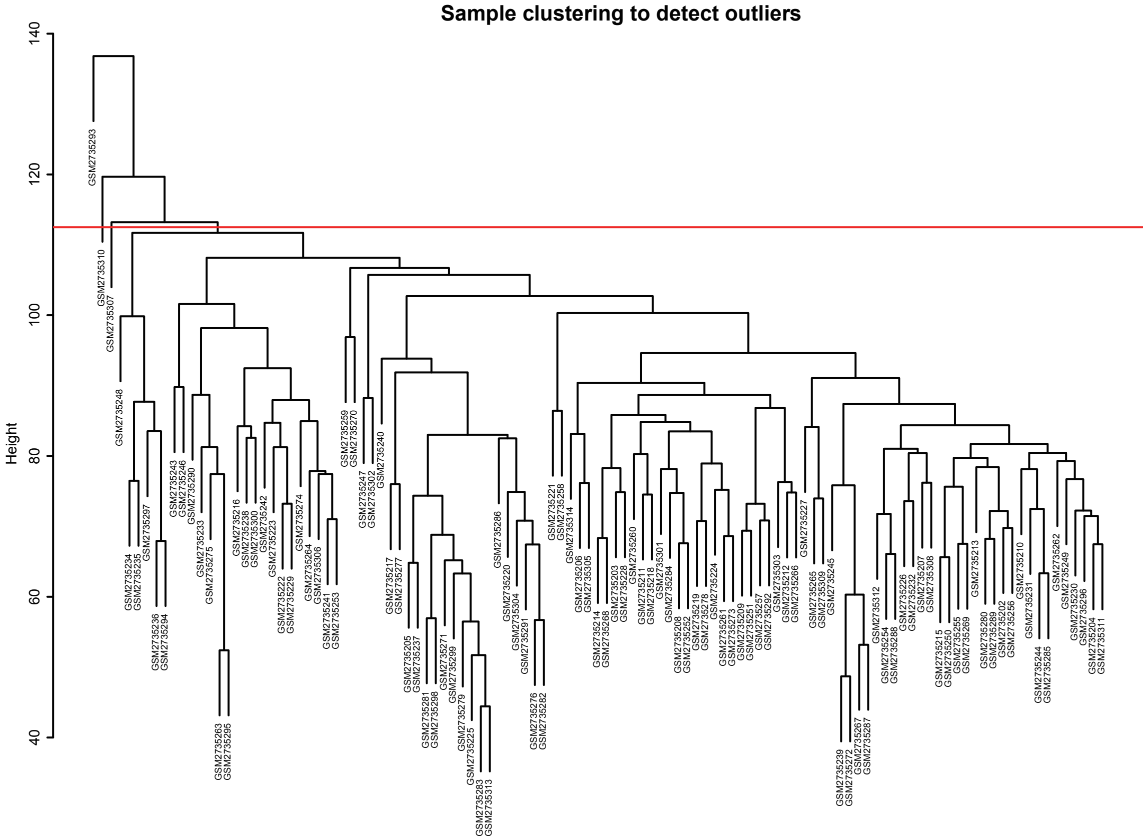

Sample clustering to detect outliers
Height
140
120
100
80
60
40
GSM2735293
GSM2735310
GSM2735307
GSM2735248
GSM2735234
GSM2735235
GSM2735297
GSM2735236
GSM2735294
GSM2735243
GSM2735246
GSM2735290
GSM2735233
GSM2735275
GSM2735263
GSM2735295
GSM2735216
GSM2735238
GSM2735300
GSM2735242
GSM2735223
GSM2735222
GSM2735229
GSM2735274
GSM2735264
GSM2735306
GSM2735241
GSM2735253
GSM2735259
GSM2735270
GSM2735247
GSM2735302
GSM2735240
GSM2735217
GSM2735277
GSM2735205
GSM2735237
GSM2735281
GSM2735298
GSM2735271
GSM2735299
GSM2735279
GSM2735225
GSM2735283
GSM2735313
GSM2735286
GSM2735220
GSM2735304
GSM2735291
GSM2735276
GSM2735282
GSM2735221
GSM2735258
GSM2735314
GSM2735206
GSM2735305
GSM2735214
GSM2735268
GSM2735203
GSM2735228
GSM2735260
GSM2735211
GSM2735218
GSM2735301
GSM2735284
GSM2735208
GSM2735252
GSM2735219
GSM2735278
GSM2735224
GSM2735261
GSM2735273
GSM2735209
GSM2735251
GSM2735257
GSM2735292
GSM2735303
GSM2735212
GSM2735266
GSM2735227
GSM2735265
GSM2735309
GSM2735245
GSM2735239
GSM2735272
GSM2735267
GSM2735287
GSM2735312
GSM2735254
GSM2735288
GSM2735226
GSM2735232
GSM2735207
GSM2735308
GSM2735215
GSM2735250
GSM2735255
GSM2735269
GSM2735213
GSM2735280
GSM2735289
GSM2735202
GSM2735256
GSM2735210
GSM2735231
GSM2735244
GSM2735285
GSM2735262
GSM2735249
GSM2735230
GSM2735296
GSM2735204
GSM2735311

Figure S3

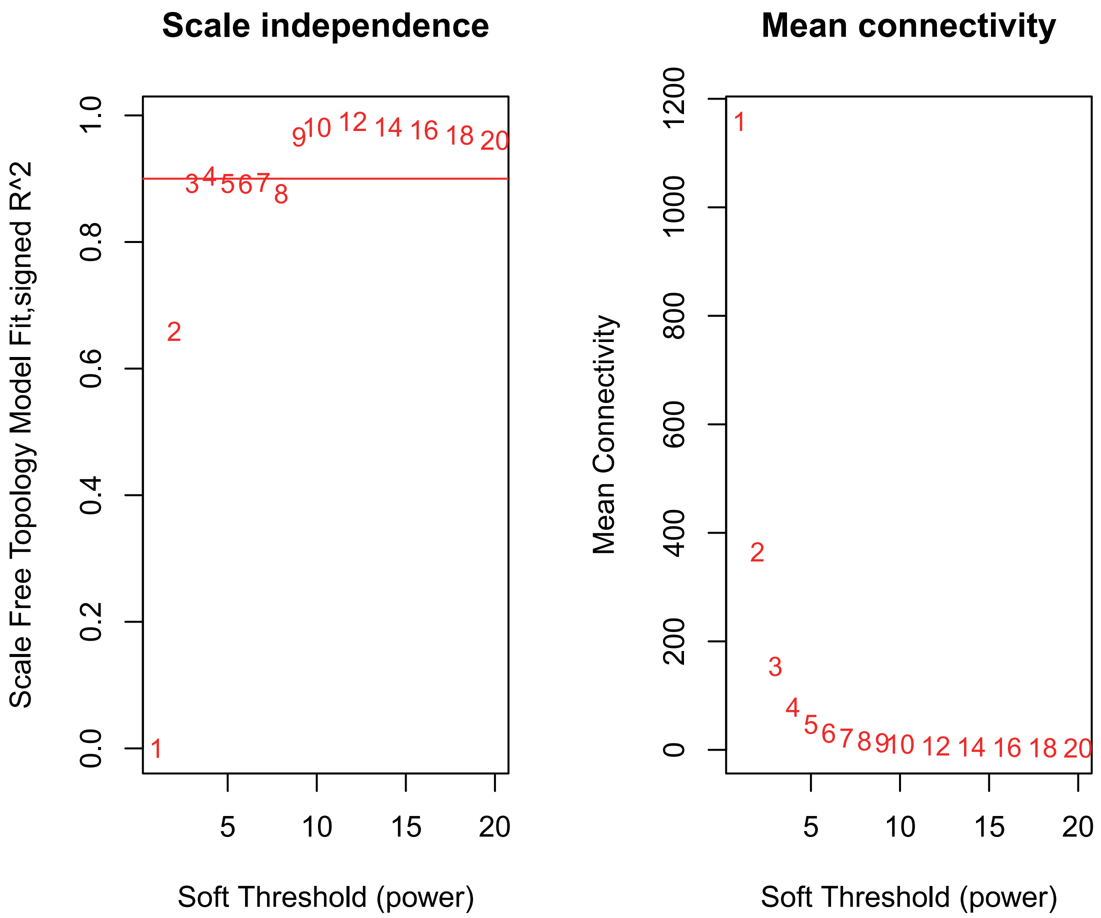

Scale independence
Scale Free Topology Model Fit,signed R^2
Soft Threshold (power)
Mean connectivity
Mean Connectivity
Soft Threshold (power)

Figure S4

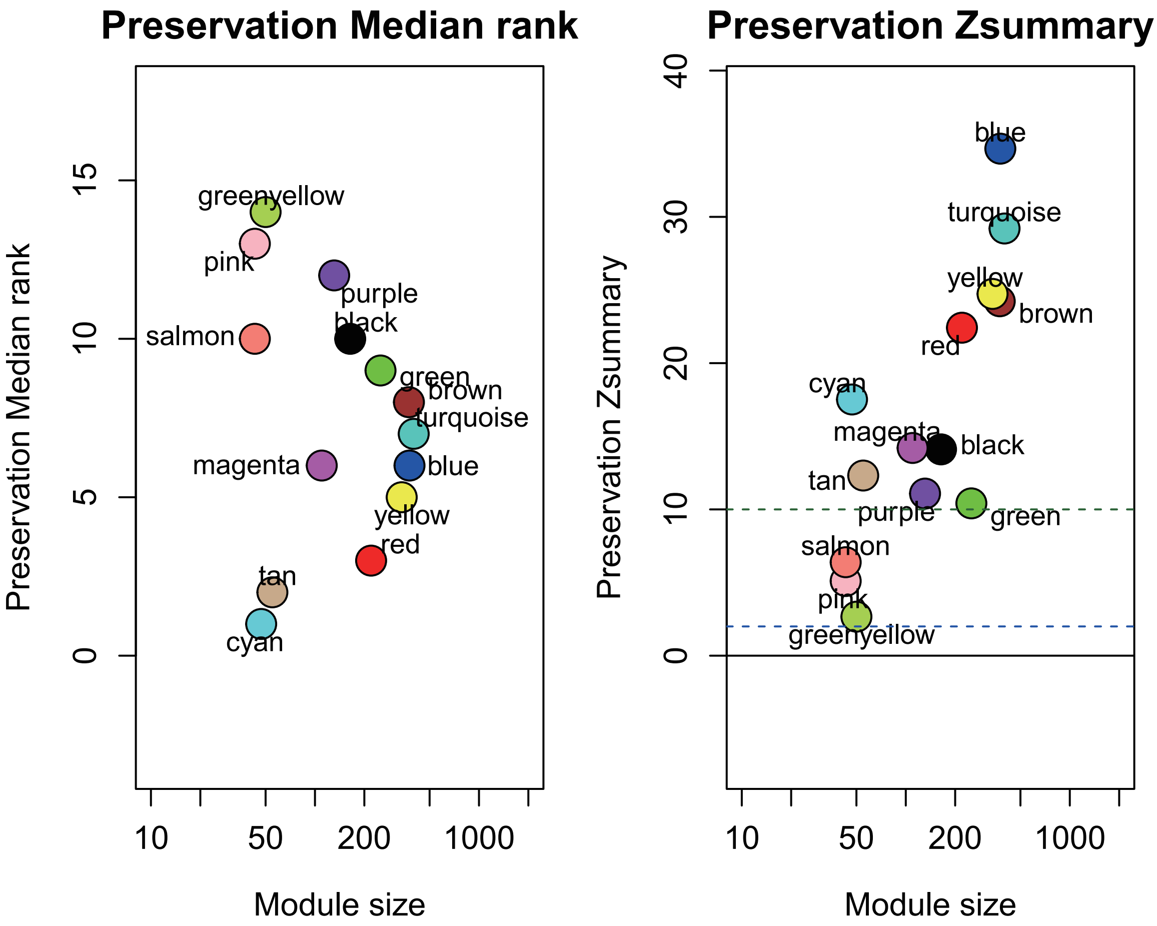

Preservation Median rank
Preservation Zsummary
Preservation Median rank
Preservation Zsummary
Module size
Module size
10
50
200
1000
0
5
10
15
0
10
20
30
40
greenyellow
pink
purple
black
salmon
green
brown
turquoise
magenta
blue
yellow
red
tan
cyan
blue
turquoise
yellow
brown
red
cyan
magenta
black
tan
purple
green
salmon
pink
greenyellow

Figure S5

(a)

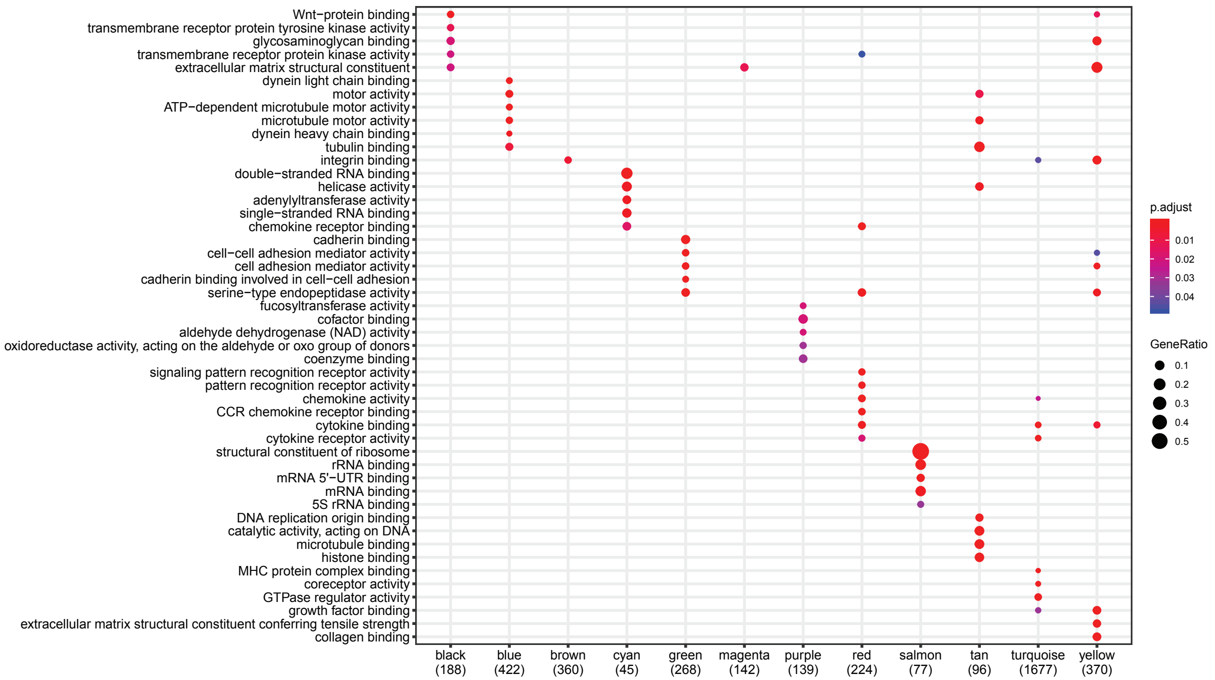

Wnt−protein binding
transmembrane receptor protein tyrosine kinase activity
glycosaminoglycan binding
transmembrane receptor protein kinase activity
extracellular matrix structural constituent
dynein light chain binding
motor activity
ATP−dependent microtubule motor activity
microtubule motor activity
dynein heavy chain binding
tubulin binding
integrin binding
double−stranded RNA binding
helicase activity
adenylyltransferase activity
single−stranded RNA binding
chemokine receptor binding
cadherin binding
cell−cell adhesion mediator activity
cell adhesion mediator activity
cadherin binding involved in cell−cell adhesion
serine−type endopeptidase activity
fucosyltransferase activity
cofactor binding
aldehyde dehydrogenase (NAD) activity
oxidoreductase activity, acting on the aldehyde or oxo group of donors
coenzyme binding
signaling pattern recognition receptor activity
pattern recognition receptor activity
chemokine activity
CCR chemokine receptor binding
cytokine binding
cytokine receptor activity
structural constituent of ribosome
rRNA binding
mRNA 5'−UTR binding
mRNA binding
5S rRNA binding
DNA replication origin binding
catalytic activity, acting on DNA
microtubule binding
histone binding
MHC protein complex binding
coreceptor activity
GTPase regulator activity
growth factor binding
extracellular matrix structural constituent conferring tensile strength
collagen binding
black (188)
blue (422)
brown (360)
cyan (45)
green (268)
magenta (142)
purple (139)
red (224)
salmon (77)
tan (96)
turquoise (1677)
yellow (370)
p.adjust
0.01
0.02
0.03
0.04
GeneRatio
0.1
0.2
0.3
0.4
0.5

(b)

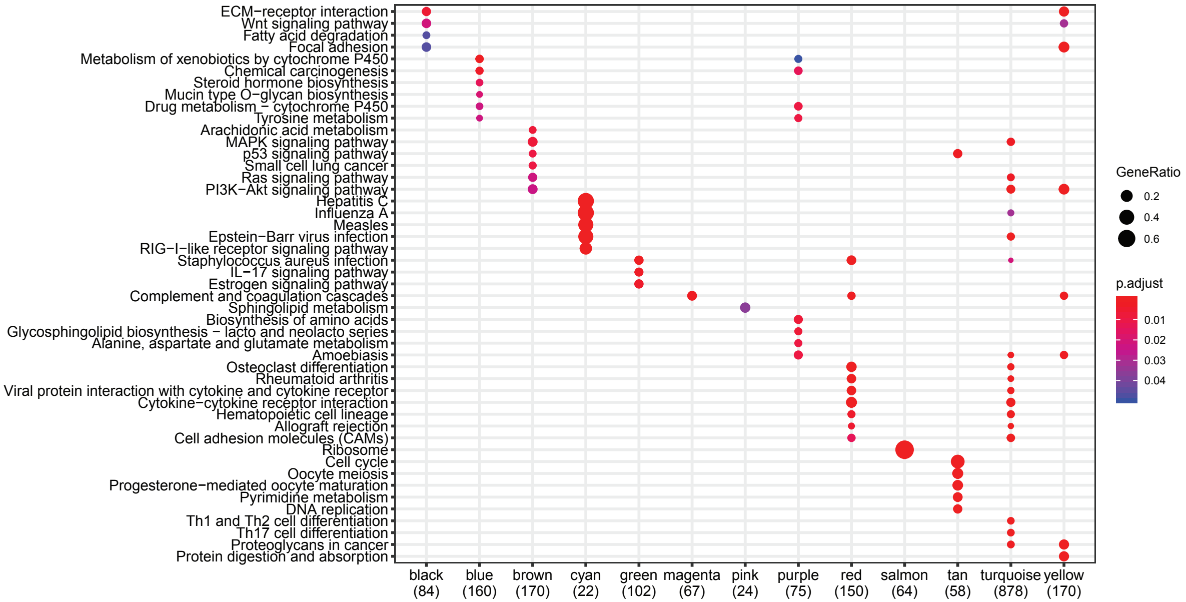

ECM−receptor interaction
Wnt signaling pathway
Fatty acid degradation
Focal adhesion
Metabolism of xenobiotics by cytochrome P450
Chemical carcinogenesis
Steroid hormone biosynthesis
Mucin type O−glycan biosynthesis
Drug metabolism − cytochrome P450
Tyrosine metabolism
Arachidonic acid metabolism
MAPK signaling pathway
p53 signaling pathway
Small cell lung cancer
Ras signaling pathway
PI3K−Akt signaling pathway
Hepatitis C
Influenza A
Measles
Epstein−Barr virus infection
RIG−I−like receptor signaling pathway
Staphylococcus aureus infection
IL−17 signaling pathway
Estrogen signaling pathway
Complement and coagulation cascades
Sphingolipid metabolism
Biosynthesis of amino acids
Glycosphingolipid biosynthesis − lacto and neolacto series
Alanine, aspartate and glutamate metabolism
Amoebiasis
Osteoclast differentiation
Rheumatoid arthritis
Viral protein interaction with cytokine and cytokine receptor
Cytokine−cytokine receptor interaction
Hematopoietic cell lineage
Allograft rejection
Cell adhesion molecules (CAMs)
Ribosome
Cell cycle
Oocyte meiosis
Progesterone−mediated oocyte maturation
Pyrimidine metabolism
DNA replication
Th1 and Th2 cell differentiation
Th17 cell differentiation
Proteoglycans in cancer
Protein digestion and absorption
black (84)
blue (160)
brown (170)
cyan (22)
green (102)
magenta (67)
pink (24)
purple (75)
red (150)
salmon (64)
tan (58)
turquoise (878)
yellow (170)
GeneRatio
0.2
0.4
0.6
p.adjust
0.01
0.02
0.03
0.04

DEmiRNAs in the ceRNA network

Symbol	logFC	AveExpr	t	P.Value	adj.P.Val	B	group	type
hsa-miR-125a-5p	-1.859889215	10.34313524	-14.46967381	3.63E-37	6.66E-35	73.71851782	downregulate	miRNA
hsa-miR-140-3p	-1.278153043	9.233931313	-11.76881169	5.60E-27	3.67E-25	50.47093093	downregulate	miRNA
hsa-miR-140-5p	-0.679776875	8.61002063	-8.87942616	4.22E-17	8.60E-16	27.99496683	downregulate	miRNA
hsa-miR-142-3p	-3.310699599	9.570592987	-16.95503002	6.11E-47	1.87E-44	96.03669615	downregulate	miRNA
hsa-miR-142-5p	-1.395608336	8.928458681	-11.81431373	3.83E-27	2.70E-25	50.84849332	downregulate	miRNA
hsa-miR-146b-5p	-0.86037352	9.433394673	-5.859195665	1.12E-08	8.12E-08	8.982019323	downregulate	miRNA
hsa-miR-28-5p	-0.557666756	8.665958377	-6.903641834	2.57E-11	2.43E-10	14.90795275	downregulate	miRNA
hsa-miR-342-3p	-1.479852746	9.262921776	-13.07559494	7.95E-32	6.63E-30	61.528846	downregulate	miRNA
hsa-miR-34c-5p	-1.443074117	8.85615501	-9.59741277	2.02E-19	5.15E-18	33.26331809	downregulate	miRNA
hsa-miR-361-3p	-0.640823507	10.13378732	-3.990416058	8.11E-05	3.38E-04	0.434521413	downregulate	miRNA
hsa-miR-361-5p	-0.717123599	8.908437512	-8.203011735	5.14E-15	7.61E-14	23.26550627	downregulate	miRNA
hsa-miR-421	-1.122229324	9.411207315	-8.098008649	1.06E-14	1.50E-13	22.55328205	downregulate	miRNA
hsa-miR-449a	-0.75204128	8.886929824	-7.840224353	6.12E-14	7.60E-13	20.83106808	downregulate	miRNA
hsa-miR-564	-0.75543993	9.61438955	-3.91195101	1.11E-04	4.40E-04	0.138770045	downregulate	miRNA
hsa-miR-574-5p	0.860834024	12.74338187	5.734293032	2.20E-08	1.54E-07	8.325333022	upregulated	miRNA

DEmRNAs in the ceRNA network

Symbol	logFC	AveExpr	t	P.Value	adj.P.Val	B	group	moduleColor
MYC	1.221070851	8.808032021	3.785372339	4.84E-04	0.004306487	-0.540282105	upregulated	salmon
NPM1	0.640885388	12.04801949	4.542466409	4.68E-05	7.02E-04	1.693775438	upregulated	salmon
CDK4	1.439838722	9.85770533	6.647218013	4.79E-08	2.45E-06	8.37858912	upregulated	turquoise
ATL2	0.874078369	7.014891468	3.761027835	5.21E-04	0.00455857	-0.609156039	upregulated	turquoise
NOP2	0.669241973	9.167533038	4.015685613	2.41E-04	0.002524064	0.121431036	upregulated	turquoise
XPOT	1.243547995	9.798126402	6.839642198	2.54E-08	1.48E-06	8.996837804	upregulated	turquoise
KIAA1549L	0.536931105	6.800755122	3.213792126	0.002527051	0.015595936	-2.09470596	upregulated	turquoise
UCK2	0.682872613	7.483707611	4.807357198	2.01E-05	3.59E-04	2.508943829	upregulated	turquoise
TFRC	0.520498319	7.554836097	5.965970222	4.54E-07	1.56E-05	6.185475301	upregulated	turquoise
TPI1	0.721345846	9.646985336	4.283292689	1.06E-04	0.001333046	0.911027683	upregulated	turquoise
GGCT	1.085088371	10.94257157	5.610064818	1.47E-06	4.27E-05	5.043618731	upregulated	turquoise
FERMT1	0.996010276	8.279014772	5.772781789	8.59E-07	2.73E-05	5.564887767	upregulated	turquoise
LAMB1	2.315728145	7.256558426	7.317460385	5.31E-09	4.07E-07	10.52459798	upregulated	turquoise
UNG	1.022193356	8.226059252	5.757386979	9.04E-07	2.84E-05	5.515505422	upregulated	turquoise
TMTC3	0.718289391	8.060036576	3.054976896	0.003911756	0.021723813	-2.500423884	upregulated	turquoise
TWSG1	0.99847215	7.561078418	3.821466852	4.35E-04	0.003961936	-0.437774991	upregulated	turquoise
FJX1	1.505847335	7.520104665	5.767304365	8.75E-07	2.76E-05	5.547316295	upregulated	turquoise
IFNG	1.163718662	6.996755616	4.618357219	3.68E-05	5.75E-04	1.925887284	upregulated	turquoise
ZNRF3	1.477360629	7.193780095	5.56070606	1.73E-06	4.85E-05	4.885832662	upregulated	turquoise
LAMC1	0.658408205	8.78349495	4.216674176	1.30E-04	0.001562873	0.712563907	upregulated	turquoise
GLCE	0.840745114	7.592239922	2.665670541	0.010879997	0.046720791	-3.43789224	upregulated	turquoise
FZD7	1.46644514	7.286328522	4.19511826	1.39E-04	0.001650514	0.64860572	upregulated	turquoise
ECT2	0.719969628	6.881106107	5.328127671	3.71E-06	9.13E-05	4.145017172	upregulated	turquoise
PHB2	0.684971543	10.86247757	3.272978957	0.002141595	0.013713934	-1.94034929	upregulated	turquoise
TM7SF3	1.138785743	8.636023627	4.240006555	1.21E-04	0.00148122	0.78193723	upregulated	turquoise
BLMH	0.713112045	8.075104735	4.112979338	1.79E-04	0.001995607	0.406098924	upregulated	turquoise
PTPN14	0.644201499	7.204456877	3.05289499	0.003933944	0.021818582	-2.505657764	upregulated	turquoise
AHCY	0.921096337	9.344512483	4.441215093	6.44E-05	9.00E-04	1.386069753	upregulated	turquoise
VANGL2	0.986451529	7.520729116	4.208124298	1.34E-04	0.001599242	0.68718029	upregulated	turquoise
ZNF738	0.739737575	6.845256784	2.657180062	0.011116338	0.047470734	-3.457362522	upregulated	turquoise
CPS1	0.609868409	5.540628317	2.666567083	0.010855313	0.046691334	-3.435833744	upregulated	turquoise
FNDC3B	0.756511446	7.430682517	4.836731582	1.83E-05	3.32E-04	2.600153151	upregulated	turquoise

SOX4	0.735307863	7.942177378	4.031773313	2.30E-04	0.002429249	0.168301442	upregulated	turquoise
IGF2BP3	0.941337942	6.459476325	3.357402424	0.001687227	0.011419083	-1.717348556	upregulated	turquoise
JAK2	0.774128797	7.099387187	3.128669031	0.003198139	0.018692305	-2.313733639	upregulated	turquoise
LDHA	1.020697726	12.39917782	5.63603087	1.35E-06	3.99E-05	5.126692764	upregulated	turquoise
LMNB2	0.969277056	7.946167817	5.464283149	2.37E-06	6.30E-05	4.578130301	upregulated	turquoise
FKBP5	0.618867401	8.04032831	5.24889518	4.80E-06	1.13E-04	3.89383255	upregulated	turquoise
PAX5	-0.948595788	7.2846298	-4.453384231	6.20E-05	8.77E-04	1.422927947	downregulate	turquoise
PRKCB	-0.547731869	7.003481117	-3.150808437	0.003008991	0.017865988	-2.257110854	downregulate	turquoise

Enriched GO terms and KEGG pathways for the genes in the ceRNA network

ONTOLOGY	ID	Description	GeneRatio	BgRatio	pvalue	p.adjust	qvalue	geneID	Count
BP	GO:0006913	nucleocytoplasmic transport	8/39	357/18493	6.51E-07	0.000550687	0.00036991	NPM1/XPOT/FERMT1/IFNG/ECT2/PHB2/PTPN14/JAK2	8
BP	GO:0051169	nuclear transport	8/39	360/18493	6.93E-07	0.000550687	0.00036991	NPM1/XPOT/FERMT1/IFNG/ECT2/PHB2/PTPN14/JAK2	8
BP	GO:0046822	regulation of nucleocytoplasmic transport	5/39	112/18493	3.64E-06	0.001927288	0.001294605	FERMT1/IFNG/ECT2/PTPN14/JAK2	5
BP	GO:0042306	regulation of protein import into nucleus	4/39	64/18493	9.80E-06	0.003738317	0.002511115	FERMT1/IFNG/ECT2/JAK2	4
BP	GO:1904589	regulation of protein import	4/39	67/18493	1.18E-05	0.003738317	0.002511115	FERMT1/IFNG/ECT2/JAK2	4
BP	GO:0006606	protein import into nucleus	5/39	157/18493	1.89E-05	0.004997154	0.003356705	FERMT1/IFNG/ECT2/PHB2/JAK2	5
BP	GO:0051170	import into nucleus	5/39	178/18493	3.45E-05	0.007829094	0.005258986	FERMT1/IFNG/ECT2/PHB2/JAK2	5
BP	GO:0000060	protein import into nucleus, translocation	3/39	38/18493	6.95E-05	0.012775392	0.008581529	IFNG/PHB2/JAK2	3
BP	GO:2000027	regulation of organ morphogenesis	5/39	208/18493	7.24E-05	0.012775392	0.008581529	MYC/ZNRF3/FZD7/PHB2/VANGL2	5
BP	GO:0017038	protein import	5/39	214/18493	8.28E-05	0.01281893	0.008610775	FERMT1/IFNG/ECT2/PHB2/JAK2	5
BP	GO:0042307	positive regulation of protein import into nucleus	3/39	42/18493	9.40E-05	0.01281893	0.008610775	IFNG/ECT2/JAK2	3
BP	GO:0000096	sulfur amino acid metabolic process	3/39	44/18493	0.000108175	0.01281893	0.008610775	BLMH/AHCY/CPS1	3
BP	GO:1904591	positive regulation of protein import	3/39	44/18493	0.000108175	0.01281893	0.008610775	IFNG/ECT2/JAK2	3
BP	GO:1900180	regulation of protein localization to nucleus	4/39	121/18493	0.00012015	0.01281893	0.008610775	FERMT1/IFNG/ECT2/JAK2	4
BP	GO:0033157	regulation of intracellular protein transport	5/39	232/18493	0.000121009	0.01281893	0.008610775	FERMT1/IFNG/ECT2/PTPN14/JAK2	5
BP	GO:1905330	regulation of morphogenesis of an epithelium	4/39	134/18493	0.000178072	0.01688805	0.011344097	ZNRF3/FZD7/PHB2/VANGL2	4
BP	GO:0051222	positive regulation of protein transport	6/39	402/18493	0.000180678	0.01688805	0.011344097	FERMT1/IFNG/ECT2/TM7SF3/SOX4/JAK2	6
BP	GO:0050673	epithelial cell proliferation	6/39	420/18493	0.000228936	0.019151203	0.012864311	MYC/FERMT1/LAMB1/LAMC1/FZD7/PHB2	6
BP	GO:0032386	regulation of intracellular transport	6/39	422/18493	0.000234871	0.019151203	0.012864311	FERMT1/IFNG/ECT2/PTPN14/JAK2/PRKCB	6
BP	GO:0034504	protein localization to nucleus	5/39	269/18493	0.000241047	0.019151203	0.012864311	FERMT1/IFNG/ECT2/PHB2/JAK2	5
BP	GO:0033160	positive regulation of protein import into nucleus, translocation	2/39	12/18493	0.000282232	0.020466883	0.013748084	IFNG/JAK2	2
BP	GO:1904951	positive regulation of establishment of protein localization	6/39	437/18493	0.000283368	0.020466883	0.013748084	FERMT1/IFNG/ECT2/TM7SF3/SOX4/JAK2	6
BP	GO:0060071	Wnt signaling pathway, planar cell polarity pathway	3/39	63/18493	0.000315495	0.021170514	0.014220729	ZNRF3/FZD7/VANGL2	3
BP	GO:0090068	positive regulation of cell cycle process	5/39	286/18493	0.000319756	0.021170514	0.014220729	NPM1/CDK4/ECT2/PHB2/SOX4	5
BP	GO:0090175	regulation of establishment of planar polarity	3/39	65/18493	0.000346019	0.021992943	0.014773174	ZNRF3/FZD7/VANGL2	3
BP	GO:0046824	positive regulation of nucleocytoplasmic transport	3/39	67/18493	0.000378384	0.022840552	0.015342532	IFNG/ECT2/JAK2	3
BP	GO:0050667	homocysteine metabolic process	2/39	14/18493	0.000388103	0.022840552	0.015342532	BLMH/CPS1	2
BP	GO:0032024	positive regulation of insulin secretion	3/39	70/18493	0.000430487	0.024051191	0.016155747	TM7SF3/SOX4/JAK2	3
BP	GO:0050796	regulation of insulin secretion	4/39	172/18493	0.000461019	0.024051191	0.016155747	IFNG/TM7SF3/SOX4/JAK2	4
BP	GO:0002009	morphogenesis of an epithelium	6/39	479/18493	0.00046207	0.024051191	0.016155747	MYC/ZNRF3/FZD7/PHB2/VANGL2/SOX4	6
BP	GO:0030111	regulation of Wnt signaling pathway	5/39	311/18493	0.000469218	0.024051191	0.016155747	FERMT1/ZNRF3/FZD7/VANGL2/SOX4	5
BP	GO:0033158	regulation of protein import into nucleus, translocation	2/39	16/18493	0.000510421	0.025345602	0.017025233	IFNG/JAK2	2
BP	GO:0007369	gastrulation	4/39	181/18493	0.000558702	0.025666543	0.017240816	LAMB1/TWSG1/FZD7/VANGL2	4
BP	GO:0046209	nitric oxide metabolic process	3/39	77/18493	0.000569435	0.025666543	0.017240816	IFNG/CPS1/JAK2	3
BP	GO:1900182	positive regulation of protein localization to nucleus	3/39	78/18493	0.000591351	0.025666543	0.017240816	IFNG/ECT2/JAK2	3
BP	GO:0034404	nucleobase-containing small molecule biosynthetic process	4/39	184/18493	0.000594303	0.025666543	0.017240816	UCK2/TPI1/UNG/LDHA	4
BP	GO:0001736	establishment of planar polarity	3/39	79/18493	0.0006138	0.025666543	0.017240816	ZNRF3/FZD7/VANGL2	3
BP	GO:0007164	establishment of tissue polarity	3/39	79/18493	0.0006138	0.025666543	0.017240816	ZNRF3/FZD7/VANGL2	3
BP	GO:2001057	reactive nitrogen species metabolic process	3/39	80/18493	0.000636787	0.025945001	0.017427863	IFNG/CPS1/JAK2	3
BP	GO:0031589	cell-substrate adhesion	5/39	338/18493	0.000684388	0.027187326	0.018262362	FERMT1/LAMB1/LAMC1/FZD7/JAK2	5
BP	GO:0034446	substrate adhesion-dependent cell spreading	3/39	87/18493	0.000813199	0.030994021	0.020819408	LAMB1/LAMC1/FZD7	3
BP	GO:0030073	insulin secretion	4/39	203/18493	0.000858236	0.030994021	0.020819408	IFNG/TM7SF3/SOX4/JAK2	4
BP	GO:0050679	positive regulation of epithelial cell proliferation	4/39	203/18493	0.000858236	0.030994021	0.020819408	MYC/LAMB1/LAMC1/FZD7	4
BP	GO:0090276	regulation of peptide hormone secretion	4/39	203/18493	0.000858236	0.030994021	0.020819408	IFNG/TM7SF3/SOX4/JAK2	4
BP	GO:0006790	sulfur compound metabolic process	5/39	362/18493	0.000931411	0.032889169	0.022092423	GGCT/GLCE/BLMH/AHCY/CPS1	5
BP	GO:0090277	positive regulation of peptide hormone secretion	3/39	93/18493	0.000986923	0.033699295	0.022636604	TM7SF3/SOX4/JAK2	3
BP	GO:0050678	regulation of epithelial cell proliferation	5/39	368/18493	0.001002398	0.033699295	0.022636604	MYC/LAMB1/LAMC1/FZD7/PHB2	5

BP	GO:0060993	kidney morphogenesis	3/39	94/18493	0.001017977	0.033699295	0.022636604	MYC/VANGL2/SOX4	3
BP	GO:0001738	morphogenesis of a polarized epithelium	3/39	95/18493	0.001049645	0.033832187	0.022725871	ZNRF3/FZD7/VANGL2	3
BP	GO:1903532	positive regulation of secretion by cell	5/39	373/18493	0.001064575	0.033832187	0.022725871	FERMT1/IFNG/TM7SF3/SOX4/JAK2	5
BP	GO:0048103	somatic stem cell division	2/39	24/18493	0.001161526	0.034947931	0.023475342	FZD7/VANGL2	2
BP	GO:0050855	regulation of B cell receptor signaling pathway	2/39	24/18493	0.001161526	0.034947931	0.023475342	PAX5/PRKCB	2
BP	GO:1901989	positive regulation of cell cycle phase transition	3/39	100/18493	0.001217343	0.034947931	0.023475342	NPM1/CDK4/PHB2	3
BP	GO:0072527	pyrimidine-containing compound metabolic process	3/39	101/18493	0.001252787	0.034947931	0.023475342	UCK2/UNG/CPS1	3
BP	GO:0015012	heparan sulfate proteoglycan biosynthetic process	2/39	25/18493	0.001260849	0.034947931	0.023475342	GLCE/VANGL2	2
BP	GO:0060330	regulation of response to interferon-gamma	2/39	25/18493	0.001260849	0.034947931	0.023475342	IFNG/JAK2	2
BP	GO:0060334	regulation of interferon-gamma-mediated signaling pathway	2/39	25/18493	0.001260849	0.034947931	0.023475342	IFNG/JAK2	2
BP	GO:0035567	non-canonical Wnt signaling pathway	3/39	102/18493	0.001288876	0.034947931	0.023475342	ZNRF3/FZD7/VANGL2	3
BP	GO:0045787	positive regulation of cell cycle	5/39	390/18493	0.001297626	0.034947931	0.023475342	NPM1/CDK4/ECT2/PHB2/SOX4	5
BP	GO:0045830	positive regulation of isotype switching	2/39	27/18493	0.001471272	0.038664814	0.025972059	TFRC/UNG	2
BP	GO:0060828	regulation of canonical Wnt signaling pathway	4/39	236/18493	0.001497839	0.038664814	0.025972059	FERMT1/ZNRF3/FZD7/SOX4	4
BP	GO:0051047	positive regulation of secretion	5/39	404/18493	0.001516363	0.038664814	0.025972059	FERMT1/IFNG/TM7SF3/SOX4/JAK2	5
BP	GO:0030099	myeloid cell differentiation	5/39	405/18493	0.001532966	0.038664814	0.025972059	MYC/TFRC/IFNG/JAK2/PRKCB	5
BP	GO:0007050	cell cycle arrest	4/39	241/18493	0.001617493	0.040159326	0.026975958	MYC/CDK4/IFNG/SOX4	4
BP	GO:0030072	peptide hormone secretion	4/39	244/18493	0.001692402	0.041372725	0.027791026	IFNG/TM7SF3/SOX4/JAK2	4
BP	GO:0050714	positive regulation of protein secretion	4/39	246/18493	0.001743665	0.041980045	0.028198977	FERMT1/TM7SF3/SOX4/JAK2	4
BP	GO:0030201	heparan sulfate proteoglycan metabolic process	2/39	31/18493	0.001938776	0.045081409	0.030282235	GLCE/VANGL2	2
BP	GO:1902751	positive regulation of cell cycle G2/M phase transition	2/39	31/18493	0.001938776	0.045081409	0.030282235	NPM1/CDK4	2
BP	GO:0001704	formation of primary germ layer	3/39	118/18493	0.001957594	0.045081409	0.030282235	LAMB1/TWSG1/FZD7	3
BP	GO:0051091	positive regulation of DNA binding transcription factor activity	4/39	255/18493	0.001987783	0.045122679	0.030309958	NPM1/PHB2/JAK2/PRKCB	4
BP	GO:0045727	positive regulation of translation	3/39	120/18493	0.002053743	0.045963349	0.030874655	NPM1/CDK4/SOX4	3
BP	GO:0050708	regulation of protein secretion	5/39	438/18493	0.002160125	0.047084881	0.031628015	FERMT1/IFNG/TM7SF3/SOX4/JAK2	5
BP	GO:0046883	regulation of hormone secretion	4/39	261/18493	0.002163119	0.047084881	0.031628015	IFNG/TM7SF3/SOX4/JAK2	4
BP	GO:0002793	positive regulation of peptide secretion	4/39	265/18493	0.00228579	0.049082708	0.032970002	FERMT1/TM7SF3/SOX4/JAK2	4
BP	GO:0010922	positive regulation of phosphatase activity	2/39	34/18493	0.002329727	0.049359158	0.0331557	IFNG/JAK2	2
CC	GO:0034399	nuclear periphery	4/42	134/19659	0.000188963	0.01757358	0.015713784	XPOT/PHB2/JAK2/LMNB2	4
MF	GO:0043024	ribosomal small subunit binding	2/38	16/17632	0.000532491	0.047309582	0.038063358	NPM1/UNG	2
MF	GO:0035173	histone kinase activity	2/38	17/17632	0.00060267	0.047309582	0.038063358	JAK2/PRKCB	2

Gene set enrichment analysis (GSEA) for ZNF667-AS1 using GSE102349

ID	setSize	enrichmentScore	NES	pvalue	p.adjust	qvalues	rank	leading_edge	core_enrichment
KEGG_CYTOKINE_CYTOKINE_RECEPTOR_INTERACTION	260	0.612102228	1.9795786	0.001067236	0.011027659	0.007341315	3261	tags=41%, list=13%, signal=36%	CCL21/CCL14/CXCL14/TNFRSF13B/CXCR5/CCL19/CXCL12/TGFBR2/TNFRSF17/CCR7/PDGFRB/TNFRSF13C/IL1R1/CCR6/CD27/CXCL13/IL18/CCL28/TNFSF12/TNFSF13B/CCL24/CD40LG/TGFB3/IL10RA/TNFSF8/IL7R/LTB/CCR2/CXCL9/TNFSF11/CXCR3/LTA/PLEKHO2/CCL11/IL21R/CSF1R/IL2RB/IL3RA/TGFB1/TNFSF13/CCR4/IL6R/IL12RB1/CXCR6/TNFRSF1B/VEGFC/ACVRL1/FLT3/CSF2RA/FLT3LG/CCL15/CCL18/TNFSF14/CSF3R/LEPR/CCR5/CCL4L2/CCR1/CCL13/IL2RG/IFNLR1/IL5RA/CLCF1/IL9R/KDR/FLT4/IL2RA/XCL2/IFNAR2/PDGFC/CXCR4/INHBB/CXCR2/XCR1/CCL2/CXCL16/TNFRSF6B/TNFRSF19/TNFRSF10D/CSF2RB/TNFRSF10C/IL6ST/CCL22/CXCL11/CCL3L1/IL4R/CCL26/IL18RAP/IFNAR1/IL18R1/CCL23/CCL3L3/TNFRSF14/TNFRSF25/CCL5/FASLG/IL21/HGF/PDGFB/PDGFRA/TNFRSF18/IL10RB/CX3CR1/TGFBR1/CCL17/CNTF
KEGG_OLFACTORY_TRANSDUCTION	249	0.478581809	1.542215299	0.001072961	0.011027659	0.007341315	3729	tags=4%, list=15%, signal=4%	PRKACB/CALM1/CLCA4/ARRB2/CLCA2/CNGA4/PRKG1/GNAL/CAMK2D/GUCA1A/OR52N4
KEGG_CHEMOKINE_SIGNALING_PATHWAY	184	0.639644324	2.016701669	0.001114827	0.011027659	0.007341315	3240	tags=43%, list=13%, signal=38%	CCL21/CCL14/CXCL14/RASGRP2/CXCR5/CCL19/CXCL12/CCR7/PRKCB/NCF1/CCR6/CXCL13/CCL28/WAS/CCL24/PLCB2/GNG11/PREX1/GNG7/PIK3CD/ITK/CCR2/FGR/VAV1/CXCL9/ADCY7/PIK3CG/ELMO1/GNB4/PRKACB/LYN/CXCR3/DOCK2/PIK3R5/CCL11/ADCY4/CCR4/CXCR6/ARRB2/CSK/GNAI2/GRK5/PTK2B/CCL15/SHC2/CCL18/GNG2/STAT5B/PRKCD/RAC2/CCR5/CCL4L2/CCR1/CCL13/GNGT2/MAPK3/RAP1A/STAT1/XCL2/CXCR4/GRK6/CXCR2/XCR1/CCL2/CXCL16/PIK3R1/CCL22/CXCL11/CCL3L1/SOS2/VAV3/CCL26/CCL23/CCL3L3/CCL5/GNG10/ROCK1/RHOA/CX3CR1/CCL17
KEGG_CALCIUM_SIGNALING_PATHWAY	176	0.504091104	1.588369872	0.001119821	0.011027659	0.007341315	3465	tags=30%, list=14%, signal=26%	ATP2A3/PLCG2/PDGFRB/PRKCB/P2RX5/CD38/MYLK/PLCB2/GNA14/F2R/CYSLTR1/ADCY7/EDNRA/PRKACB/ADCY4/CALM1/EDNRB/PPP3CC/P2RX1/BST1/ITPR2/PTK2B/PLCD1/PTGER3/PTAFR/ITPR1/NOS3/P2RX7/SLC8A1/PTGFR/PLCE1/GNAL/SPHK1/CAMK2D/PDE1B/HRH1/OXTR/PRKCA/TNNC1/CACNA1C/CAMK4/NOS2/ADORA2A/TRPC1/GNAQ/PDGFRA/TNNC2/PDE1A/LTB4R2/TBXA2R/PPP3CA/ADRB2/CACNA1H
KEGG_NATURAL_KILLER_CELL_MEDIATED_CYTOTOXICITY	132	0.601581433	1.839628229	0.001176471	0.011027659	0.007341315	3760	tags=43%, list=15%, signal=37%	CD48/PLCG2/PRKCB/NCR3/ITGAL/KLRK1/ITGB2/HCST/PIK3CD/VAV1/PTPN6/LCK/CD247/PIK3CG/LAT/HLA-E/SH2D1A/FYN/PIK3R5/ZAP70/LCP2/TYROBP/HLA-C/NFATC2/PPP3CC/PTK2B/HLA-G/NFATC1/NFATC4/SHC2/HLA-B/RAC2/MAPK3/RAET1E/FCER1G/HLA-A/IFNAR2/KLRD1/CD244/MICB/TNFRSF10D/PIK3R1/FCGR3A/ICAM2/TNFRSF10C/SOS2/VAV3/PRKCA/KLRC3/IFNAR1/FASLG/KLRC1/PPP3CA/RAET1G/GRB2/SHC1/SYK
KEGG_SYSTEMIC_LUPUS_ERYTHEMATOSUS	124	0.537187019	1.634244652	0.001184834	0.011027659	0.007341315	3653	tags=27%, list=15%, signal=23%	C3/C1S/C7/C2/CD40LG/CTSG/HLA-DQA2/CD28/FCGR2B/C1QA/C1QC/C1R/CD86/HLA-DOB/C1QB/ACTN1/HLA-DMA/HLA-DMB/HLA-DOA/HLA-DPA1/FCGR2C/HIST2H2BE/FCGR1A/HLA-DPB1/H2AFJ/HLA-DRA/FCGR3A/CD80/HIST2H2AA4/HIST2H4A/HLA-DRB1/TRIM21/HIST1H2AC/FCGR2A
KEGG_CELL_ADHESION_MOLECULES_CAMS	128	0.615629301	1.872884903	0.001190476	0.011027659	0.007341315	3135	tags=48%, list=13%, signal=42%	CD22/SELL/SELP/ITGA4/PTPRC/CD6/ITGAL/ICAM3/CD40LG/CADM3/ITGB2/HLA-DQA2/JAM3/CD28/PECAM1/JAM2/SELPLG/CD4/CD2/CD34/HLA-E/CD86/CD8B/ITGB7/CDH5/SPN/ICOS/HLA-DOB/CD8A/PDCD1/HLA-C/ESAM/HLA-DMA/HLA-DMB/HLA-DOA/HLA-DPA1/HLA-G/CLDN23/CLDN10/CD226/HLA-B/CLDN11/CTLA4/CLDN5/HLA-F/ITGA8/PDCD1LG2/HLA-A/VCAN/HLA-DPB1/PTPRM/SIGLEC1/HLA-DRA/NFASC/ICAM2/NEGR1/CD80/CLDN3/SDC2/CD99/CDH2/HLA-DRB1
KEGG_LYSOSOME	119	0.574686245	1.735776254	0.001201923	0.011027659	0.007341315	4695	tags=50%, list=19%, signal=40%	CTSK/FUCA1/LAPTM5/CTSG/CTSS/CD68/CTSZ/ACP5/GALC/CTSC/CTSF/CTSH/LGMN/AP1S2/IDS/CTSB/ATP6V0D1/LIPA/SMPD1/CTSD/TPP1/NAGA/TCIRG1/NPC2/SLC17A5/CTSA/CTSW/PLA2G15/PSAP/GBA/AP1M2/HEXA/CTNS/ARSA/GNPTG/PPT1/AP4S1/HYAL1/ATP6V0A4/GM2A/ATP6AP1/ARSG/ACP2/GNPTAB/CTSO/GALNS/AP1G1/NEU1/HEXB/GLB1/LAPTM4A/ATP6V0A1/GNS/AP4B1/MAN2B1/ASAH1/ATP6V0D2/ATP6V0C/SORT1

KEGG_T_CELL_RECEPTOR_SIGNALING_PATHWAY	107	0.566725816	1.692478382	0.001218027	0.011027659	0.007341315	3753	tags=44%, list=15%, signal=37%	PTPRC/CARD11/CD3E/CD40LG/CD28/PIK3CD/ITK/CD3D/CD3G/VAV1/PTPN6/CD4/LCK/CD247/PIK3CG/LAT/CD8B/FYN/ICOS/PIK3R5/ZAP70/CD8A/LCP2/GRAP2/PDCD1/NFATC2/PPP3CC/PRKCQ/NFATC1/NFATC4/CTLA4/RASGRP1/FOS/MAPK3/MAP3K8/CBL/PIK3R1/SOS2/VAV3/TEC/RHOA/MAPK14/PPP3CA/PAK2/GRB2/IL2/CBLB
KEGG_LEUKOCYTE_TRANSENDOTHELIAL_MIGRATION	115	0.617231688	1.853976465	0.001219512	0.011027659	0.007341315	3366	tags=43%, list=14%, signal=38%	CXCL12/MMP2/PLCG2/ITGA4/MYL9/PRKCB/NCF1/RHOH/CYBB/ITGAL/ITGB2/JAM3/PIK3CD/SIPA1/ITK/PECAM1/CYBA/VAV1/JAM2/MMP9/PIK3CG/CDH5/PIK3R5/ACTN1/ESAM/THY1/TXK/GNAI2/PTK2B/CLDN23/CLDN10/CLDN11/CLDN5/RAC2/RAP1A/NCF4/VASP/CXCR4/PIK3R1/NCF2/RASSF5/VAV3/CLDN3/PRKCA/RAPGEF3/CD99/ROCK1/RHOA/MAPK14/ACTB
KEGG_VASCULAR_SMOOTH_MUSCLE_CONTRACTION	113	0.554375529	1.660843367	0.00122399	0.011027659	0.007341315	3192	tags=33%, list=13%, signal=29%	PLA2G2D/RAMP3/MYL9/PRKCB/MYLK/PLCB2/ADCY7/EDNRA/ARHGEF1/PRKACB/ACTA2/RAMP2/MYH11/ADCY4/CALM1/PLA2G2A/PRKCQ/MRVI1/ITPR2/CALCRL/PRKCH/PRKCD/MAPK3/ITPR1/NPR2/PRKG1/CALD1/PPP1R12B/PPP1R14A/PRKCA/CACNA1C/ADORA2A/ROCK1/GNAQ/NPR1/RHOA/PLA2G10
KEGG_ANTIGEN_PROCESSING_AND_PRESENTATION	80	0.610697234	1.772233527	0.001257862	0.011027659	0.007341315	3175	tags=35%, list=13%, signal=31%	HLA-DQA2/CTSS/CD4/HLA-E/CD8B/LTA/HLA-DOB/CD8A/LGMN/HLA-C/HLA-DMA/HLA-DMB/HLA-DOA/CTSB/HLA-DPA1/HLA-G/HLA-B/B2M/HLA-F/IFI30/HLA-A/HLA-DPB1/KLRD1/CD74/HLA-DRA/KLRC3/HLA-DRB1/KLRC1
KEGG_HEMATOPOIETIC_CELL_LINEAGE	84	0.73632525	2.14111201	0.001262626	0.011027659	0.007341315	2599	tags=54%, list=11%, signal=48%	MS4A1/CR2/CD22/CD19/CD37/ITGA4/IL1R1/CD38/CR1/FCER2/CD5/CD3E/IL7R/CD3D/CD3G/CD1C/CD4/CD55/CD2/CD34/ITGA1/CD8B/GP1BA/CD1D/CD7/CD8A/CSF1R/IL3RA/IL6R/CD14/FLT3/CSF2RA/FLT3LG/CSF3R/MME/CD1E/IL5RA/ITGA5/IL9R/FCGR1A/IL2RA/CD36/CD33/HLA-DRA/IL4R
KEGG_B_CELL_RECEPTOR_SIGNALING_PATHWAY	75	0.668235344	1.927123046	0.001264223	0.011027659	0.007341315	2919	tags=45%, list=12%, signal=40%	CD79A/CR2/CD22/CD19/CD79B/CD72/PLCG2/BTK/PRKCB/CARD11/RASGRP3/PIK3AP1/PIK3CD/VAV1/FCGR2B/PTPN6/PIK3CG/LYN/PIK3R5/NFATC2/PPP3CC/IFITM1/INPP5D/NFATC1/NFATC4/RAC2/FOS/MAPK3/PIK3R1/SOS2/VAV3/LILRB3/CD81/BLNK
KEGG_LEISHMANIA_INFECTION	70	0.629671665	1.799886943	0.001277139	0.011027659	0.007341315	3295	tags=47%, list=13%, signal=41%	C3/ITGA4/PRKCB/NCF1/CR1/TGFB3/ITGB2/HLA-DQA2/CYBA/TLR4/PTPN6/HLA-DOB/TGFB1/HLA-DMA/HLA-DMB/HLA-DOA/HLA-DPA1/FCGR2C/FOS/MAPK3/MYD88/FCGR1A/STAT1/HLA-DPB1/NCF4/HLA-DRA/NCF2/FCGR3A/JAK1/TLR2/NOS2/HLA-DRB1/MAPK14
KEGG_METABOLISM_OF_XENOBIOTICS_BY_CYTOCHROME_P450	70	0.617602495	1.765387786	0.001277139	0.011027659	0.007341315	3176	tags=31%, list=13%, signal=27%	GSTA1/ALDH1A3/ALDH3A1/AKR1C3/ADH1B/ALDH3B1/ADH1C/CYP1B1/GSTA2/ADH7/MGST1/GSTM3/CYP2F1/GSTM5/GSTM2/EPHX1/GSTK1/UGT2A1/GSTM4/GSTA3/GSTM1/GSTT1
KEGG_COMPLEMENT_AND_COAGULATION_CASCADES	68	0.793716221	2.263206549	0.00128041	0.011027659	0.007341315	1494	tags=38%, list=6%, signal=36%	CR2/C3/SERPING1/CFH/C1S/CFI/C7/F13A1/A2M/CR1/C2/PROS1/F2R/SERPINA1/MASP1/C1QA/F3/CD55/C1QC/C1R/CFB/THBD/C1QB/VWF/F10/C3AR1
KEGG_DRUG_METABOLISM_CYTOCHROME_P450	72	0.632120592	1.812924413	0.00128041	0.011027659	0.007341315	3176	tags=33%, list=13%, signal=29%	GSTA1/FMO2/ALDH1A3/ALDH3A1/MAOB/ADH1B/ALDH3B1/ADH1C/GSTA2/ADH7/MGST1/GSTM3/FMO3/FMO1/FMO5/GSTM5/GSTM2/GSTK1/AOX1/UGT2A1/GSTM4/GSTA3/GSTM1/GSTT1
KEGG_AUTOIMMUNE_THYROID_DISEASE	50	0.721516754	1.965440377	0.001321004	0.011027659	0.007341315	4983	tags=54%, list=20%, signal=43%	CD40LG/HLA-DQA2/CD28/HLA-E/CD86/HLA-DOB/HLA-C/HLA-DMA/HLA-DMB/HLA-DOA/HLA-DPA1/HLA-G/HLA-B/CTLA4/HLA-F/HLA-A/HLA-DPB1/HLA-DRA/CD80/FASLG/HLA-DRB1/IL2/PRF1/IL10/HLA-DQB1/TSHR/HLA-DQA1
KEGG_INTESTINAL_IMMUNE_NETWORK_FOR_IGA_PRODUCTION	45	0.774684005	2.082738993	0.001335113	0.011027659	0.007341315	2644	tags=58%, list=11%, signal=52%	PIGR/TNFRSF13B/CXCL12/TNFRSF17/ITGA4/TNFRSF13C/CCL28/TNFSF13B/CD40LG/HLA-DQA2/CD28/CD86/ITGB7/ICOS/HLA-DOB/TGFB1/TNFSF13/HLA-DMA/AICDA/HLA-DMB/HLA-DOA/HLA-DPA1/HLA-DPB1/CXCR4/HLA-DRA/CD80
KEGG_GRAFT_VERSUS_HOST_DISEASE	37	0.694149021	1.787917407	0.001385042	0.011027659	0.007341315	5255	tags=76%, list=21%, signal=60%	HLA-DQA2/CD28/HLA-E/CD86/HLA-DOB/HLA-C/HLA-DMA/HLA-DMB/HLA-DOA/HLA-DPA1/HLA-G/HLA-B/HLA-F/HLA-A/HLA-DPB1/KLRD1/HLA-DRA/CD80/FASLG/HLA-DRB1/KLRC1/IL2/PRF1/TNF/HLA-DQB1/HLA-DQA1/KIR3DL2/IFNG
KEGG_ALLOGRAFT_REJECTION	35	0.73183931	1.866659265	0.001396648	0.011027659	0.007341315	4983	tags=77%, list=20%, signal=62%	CD40LG/HLA-DQA2/CD28/HLA-E/CD86/HLA-DOB/HLA-C/HLA-DMA/HLA-DMB/HLA-DOA/HLA-DPA1/HLA-G/HLA-B/HLA-F/HLA-A/HLA-DPB1/HLA-DRA/CD80/FASLG/HLA-DRB1/IL2/PRF1/IL12B/IL10/TNF/HLA-DQB1/HLA-DQA1
KEGG_PRIMARY_IMMUNODEFICIENCY	35	0.783243433	1.997772723	0.001396648	0.011027659	0.007341315	1748	tags=51%, list=7%, signal=48%	CD79A/CD19/TNFRSF13B/BTK/PTPRC/TNFRSF13C/CD3E/CD40LG/IL7R/CD3D/CD4/LCK/CD8B/ICOS/ZAP70/CD8A/AICDA/IL2RG

KEGG_ASTHMA	28	0.741195043	1.818231914	0.001430615	0.011027659	0.007341315	4983	tags=64%, list=20%, signal=51%	CD40LG/HLA-DQA2/HLA-DOB/CCL11/HLA-DMA/HLA-DMB/HLA-DOA/HLA-DPA1/FCER1G/HLA-DPB1/MS4A2/HLA-DRA/PRG2/HLA-DRB1/IL10/TNF/HLA-DQB1/HLA-DQA1
KEGG_NEUROACTIVE_LIGAND_RECEPTOR_INTERACTION	266	0.468512827	1.516013339	0.002134472	0.015794416	0.010514633	5438	tags=29%, list=22%, signal=23%	S1PR1/ADRA2A/P2RY8/P2RX5/S1PR4/GABRP/P2RY10/P2RY13/CNR2/CTSG/LPAR1/F2R/CYSLTR1/EDNRA/APLNR/EDNRB/P2RY14/FPR1/P2RX1/C3AR1/CALCRL/PTGER3/PTGER2/F2RL2/LEPR/P2RY11/FPR3/PTAFR/P2RX7/F2RL1/P2RY6/GIPR/VIPR1/PTGFR/GRIN3B/CHRNA1/HRH1/OXTR/PRSS3/ADORA2A/LTB4R2/GZMA/TBXA2R/F2RL3/SSTR3/LTB4R/OPRL1/GPR35/ADRB2/CNR1/TACR1/NR3C1/PTH1R/MC1R/GRID1/PTGDR/THRA/C5AR1/CHRNB1/FPR2/GRIA3/GRIN3A/HRH2/ADORA3/GRIN2D/ADRA2C/NPY1R/GLRB/CYSLTR2/TSHR/NPFFR1/AVPR2/GRM5/AVPR1A/LPAR6/ADRB1/PTGER1
KEGG_FOCAL_ADHESION	197	0.461085845	1.459695851	0.002219756	0.015794416	0.010514633	3715	tags=37%, list=15%, signal=31%	COL6A3/COL3A1/COL1A2/PDGFRB/ITGA4/MYL9/PRKCB/PARVG/THBS2/COL1A1/COL6A2/MYLK/PIK3CD/COL5A2/VAV1/LAMA4/PIK3CG/THBS1/COL6A1/TNC/ITGA1/TNXB/ITGB7/FYN/PIK3R5/COMP/ACTN1/VWF/LAMA2/VEGFC/IGF1/SHC2/ITGA11/RAC2/COL11A1/MAPK3/ITGA5/FN1/ITGA8/RAP1A/TLN1/CCND3/KDR/FLT4/ZYX/FLNC/COL5A3/VASP/PDGFC/LAMB2/PIK3R1/PARVB/SOS2/VAV3/PRKCA/DIAPH1/COL6A6/HGF/ROCK1/PDGFB/PDGFRA/PDGFD/RHOA/PARVA/ITGB3/RAPGEF1/ACTB/PAK2/ITGA9/GRB2/BCAR1/SHC1
KEGG_MISMATCH_REPAIR	23	-0.703948891	-1.896726496	0.002873563	0.019689229	0.013107481	2467	tags=57%, list=10%, signal=51%	SSBP1/MSH2/RFC3/RFC2/LIG1/RFC4/MSH6/EXO1/POLD2/PCNA/POLD1/RFC5
KEGG_HOMOLOGOUS_RECOMBINATION	26	-0.649811876	-1.798685687	0.00304878	0.020143728	0.01341005	5259	tags=77%, list=21%, signal=60%	XRCC3/MUS81/RPA1/TOP3B/BRCA2/NBN/RAD51D/RPA3/BLM/SSBP1/RAD51C/RAD52/XRCC2/RAD54B/RAD54L/EME1/RAD51/POLD2/POLD1
KEGG_RNA_POLYMERASE	29	-0.643017111	-1.794584315	0.003344482	0.020487265	0.013638748	6125	tags=86%, list=25%, signal=65%	POLR2G/POLR2J3/POLR1E/POLR3C/POLR2B/POLR2L/POLR3F/POLR3K/POLR2J2/POLR3D/POLR2E/POLR3A/POLR2J/POLR1B/POLR2K/POLR1C/POLR1D/POLR3B/POLR3H/POLR1A/POLR2D/POLR2F/POLR2I/POLR2H
KEGG_BASE_EXCISION_REPAIR	33	-0.56956501	-1.652580776	0.003460208	0.020487265	0.013638748	4644	tags=64%, list=19%, signal=52%	APEX2/MUTYH/POLL/POLB/PARP1/POLE3/XRCC1/TDG/LIG3/SMUG1/POLE2/NEIL3/FEN1/LIG1/POLE/POLD2/NTHL1/PCNA/POLD1/UNG
KEGG_BASAL_TRANSCRIPTION_FACTORS	35	-0.640821048	-1.881168456	0.003496503	0.020487265	0.013638748	5100	tags=51%, list=21%, signal=41%	TAF4/TAF11/GTF2E2/TAF6L/GTF2F1/TAF5L/GTF2H4/GTF2A2/TAF5/GTF2I/GTF2H1/TBP/TBPL1/GTF2F2/TAF4B/GTF2H3/GTF2IRD1
KEGG_DNA_REPLICATION	36	-0.770098588	-2.266655581	0.003584229	0.020487265	0.013638748	3375	tags=75%, list=14%, signal=65%	RPA3/POLA2/RNASEH1/SSBP1/MCM5/PRIM2/POLA1/POLE2/MCM6/PRIM1/FEN1/DNA2/MCM3/RFC3/RFC2/LIG1/POLE/RFC4/MCM7/POLD2/PCNA/POLD1/RFC5/MCM2/RNASEH2A/MCM4
KEGG_AMINOACYL_TRNA_BIOSYNTHESIS	41	-0.671280404	-2.019557464	0.003831418	0.020487265	0.013638748	4960	tags=73%, list=20%, signal=58%	AARS/AARS2/TARS2/DARS2/RARS/PSTK/RARS2/VARS/DARS/CARS2/IARS2/WARS2/EPRS/SARS/CARS/YARS/PARS2/NARS/MARS2/GARS/FARSB/IARS/TARS/MARS/FARSA/LARS/YARS2/EARS2/SARS2
KEGG_NUCLEOTIDE_EXCISION_REPAIR	44	-0.613224348	-1.872606502	0.003846154	0.020487265	0.013638748	4759	tags=57%, list=19%, signal=46%	GTF2H5/RBX1/DDB1/GTF2H4/CUL4A/RAD23B/CDK7/POLE3/RPA3/ERCC3/GTF2H1/DDB2/POLE2/GTF2H3/RAD23A/RFC3/RFC2/LIG1/POLE/RFC4/POLD2/PCNA/POLD1/RFC5
KEGG_PROTEASOME	43	-0.645455604	-1.960484601	0.003875969	0.020487265	0.013638748	4686	tags=63%, list=19%, signal=51%	PSME4/PSMC2/POMP/PSMD12/PSMB5/PSMD4/PSME3/PSMA5/PSMB3/PSMC5/PSMA6/PSMA4/PSMD8/PSMB1/PSMC3/PSMB2/PSMD11/PSMB4/PSMD2/PSMA7/PSMA1/PSMA2/PSMD3/PSMB7/PSMD14/PSMC4
KEGG_RNA_DEGRADATION	52	-0.488481516	-1.551407151	0.004065041	0.020889792	0.013906718	6049	tags=54%, list=25%, signal=41%	SKIV2L/CNOT4/LSM3/CNOT7/LSM1/EXOSC9/EXOSC1/EDC3/EXOSC7/PATL1/CNOT3/WDR61/XRN2/CNOT6/LSM6/LSM7/EXOSC2/PNPT1/HSPA9/LSM2/EXOSC4/ENO1/LSM5/ENO2/LSM4/EXOSC5/HSPD1
KEGG_P53_SIGNALING_PATHWAY	65	-0.50297341	-1.663776396	0.004444444	0.022222222	0.014793741	3226	tags=45%, list=13%, signal=39%	SESN1/CDKN1A/BBC3/CCND2/DDB2/FAS/EI24/BID/TP53/SFN/CHEK1/CCNE2/MDM2/STEAP3/GTSE1/GADD45A/TNFRSF10B/CYCS/CHEK2/RRM2/TP73/CCND1/CCNB1/CDK4/PERP/PMAIP1/CDK1/CCNB2
KEGG_RIBOSOME	85	-0.795730299	-2.795456505	0.004784689	0.023293881	0.015507164	3009	tags=75%, list=12%, signal=66%	RPS15A/RPL3/RPS20/RPL30/RPL23A/RPS9/RPL28/RPL9/RPL22/RPLP2/RPS23/RPL37/RPS27A/RPL10A/UBA52/RPL39/RSL24D1/RPL26L1/RPL12/RPL19/RPS2/RPL27A/RPL23/RPL27/RPL4/RPL7A/RPL36A/RPS13/RPS3/RPS11/RPL35/RPL7/RPS10/RPS8/RPL41/RPS28/MRPL13/RPL31/RPS4X/RPLP1/RPS3A/RPL38/RPS16/RPL8/RPL13A/RPL17/RPL18/RPL36/RPS18/RPL35A/RPS26/RPS12/RPL22L1/RPL18A/RPL37A/RPS15/RPS24/RPS7/RPL24/RPS21/RPS5/RPS19/RPLP0

KEGG_VIRAL_MYOCARDITIS	68	0.547347031	1.560708164	0.005121639	0.023874048	0.015893392	4983	tags=56%, list=20%, signal=45%	ITGAL/CD40LG/ITGB2/HLA-DQA2/CD28/CD55/HLA-E/CD86/FYN/HLA-DOB/MYH11/HLA-C/LAMA2/HLA-DMA/HLA-DMB/HLA-DOA/HLA-DPA1/HLA-G/HLA-B/RAC2/HLA-F/HLA-A/HLA-DPB1/HLA-DRA/SGCB/CD80/HLA-DRB1/ACTB/SGCD/MYH10/CASP8/PRF1/EIF4G3/CASP9/SGCA/CAV1/HLA-DQB1/HLA-DQA1
KEGG_PYRIMIDINE_METABOLISM	98	-0.518307527	-1.853248488	0.005208333	0.023874048	0.015893392	3556	tags=48%, list=14%, signal=41%	POLR2J2/POLR3D/POLE3/POLR2E/ENTPD6/POLR3A/POLA2/POLR2J/CANT1/CTPS2/POLR1B/POLR2K/UCKL1/POLR1C/POLR1D/PRIM2/UMPS/POLR3B/POLA1/POLE2/PNPT1/POLR3H/POLR1A/DCTD/NME1-NME2/NME4/POLR2D/POLR2F/PRIM1/RRM1/UCK2/POLR2I/DUT/CAD/POLR2H/POLE/NT5M/RRM2/CTPS1/TYMS/POLD2/NME2/POLD1/DTYMK/NME1/TK1
KEGG_OXIDATIVE_PHOSPHORYLATION	101	-0.488252151	-1.761012739	0.005291005	0.023874048	0.015893392	5569	tags=62%, list=23%, signal=48%	NDUFB2/NDUFV2/COX8A/ATP6V1E1/COX10/COX15/NDUFS1/NDUFC1/NDUFA3/SDHB/UQCRB/UQCRC1/NDUFB6/NDUFA2/COX4I1/NDUFS7/NDUFB10/NDUFB5/NDUFS3/NDUFA1/NDUFS2/NDUFA5/UQCRC2/UQCR10/ATP6V1C2/COX7C/COX5A/COX11/NDUFA10/NDUFA6/PPA1/PPA2/NDUFA8/NDUFB3/ATP6V1E2/UQCR11/NDUFA4/NDUFAB1/NDUFS4/UQCRFS1/NDUFA11/UQCRQ/COX7A2L/COX6C/NDUFS5/COX7A2/COX17/COX5B/NDUFB4/COX7B/NDUFB7/COX6B1/NDUFA7/COX6A1/NDUFS6/NDUFS8/NDUFV1/NDUFA9/CYC1/UQCRH/NDUFB9/COX6B2
KEGG_PARKINSONS_DISEASE	99	-0.532047382	-1.888270426	0.005434783	0.023938923	0.015936581	5569	tags=65%, list=23%, signal=50%	NDUFB2/NDUFV2/COX8A/GPR37/PARK7/NDUFS1/NDUFC1/NDUFA3/SDHB/UQCRB/UQCRC1/NDUFB6/NDUFA2/COX4I1/NDUFS7/NDUFB10/NDUFB5/NDUFS3/NDUFA1/NDUFS2/NDUFA5/UQCRC2/UQCR10/PPID/VDAC3/COX7C/UBE2L3/COX5A/SLC25A4/NDUFA10/NDUFA6/NDUFA8/NDUFB3/UQCR11/SLC25A5/NDUFA4/NDUFAB1/NDUFS4/UQCRFS1/UQCRQ/COX7A2L/COX6C/NDUFS5/COX7A2/COX5B/NDUFB4/COX7B/NDUFB7/COX6B1/VDAC2/NDUFA7/COX6A1/SLC25A6/NDUFS6/VDAC1/NDUFS8/NDUFV1/NDUFA9/CYCS/CYC1/UQCRH/NDUFB9/COX6B2
KEGG_UBIQUITIN_MEDIATED_PROTEOLYSIS	130	-0.432094801	-1.598048922	0.00625	0.026353276	0.01754386	6106	tags=51%, list=25%, signal=38%	BTRC/FBXW11/STUB1/UBE2G2/UBE2J2/UBA1/HUWE1/UBE2F/RNF7/UBE2B/FBXO4/BIRC3/PIAS1/UBOX5/AIRE/UBE4B/RBX1/UBE2N/CUL1/UBE2K/ANAPC10/CUL2/DDB1/FZR1/PIAS2/UBE2W/UBE2D4/PIAS4/TRIM37/SMURF1/CUL4A/SKP1/FANCL/CDC23/UBE2L3/ANAPC1/UBE3B/UBE2O/UBE3C/SAE1/ANAPC5/CUL7/UBE3A/UBE2E3/CDC34/PRPF19/UBE2Z/FBXW8/ANAPC7/DDB2/HERC2/ANAPC11/KEAP1/UBE2M/MDM2/UBA2/BRCA1/KLHL13/NEDD4L/SKP2/CDC20/CBLC/UBE2S/SOCS1/UBE2C
KEGG_CELL_CYCLE	124	-0.603265139	-2.210454942	0.006329114	0.026353276	0.01754386	4497	tags=62%, list=18%, signal=51%	MAD1L1/CUL1/ANAPC10/HDAC1/ORC3/FZR1/SMAD4/ORC2/E2F5/SKP1/SMAD3/SMC3/CDK7/CCNE1/YWHAE/ORC4/DBF4/E2F2/CDK2/BUB3/CDC23/ANAPC1/CDKN1A/RAD21/ANAPC5/RBL1/YWHAQ/ORC5/MCM5/CDC7/SMC1A/CCND2/ANAPC7/YWHAG/BUB1/ANAPC11/TFDP1/TP53/MCM6/SFN/CHEK1/CCNE2/HDAC2/MDM2/ORC6/MCM3/GADD45A/CDC25C/PLK1/ORC1/PKMYT1/CCNA2/PRKDC/CDC25A/ESPL1/MYC/BUB1B/TTK/SKP2/WEE1/CHEK2/MCM7/CCND1/CDC20/PCNA/PTTG1/CCNB1/CDK4/MCM2/MCM4/E2F1/CDK1/CCNB2/MAD2L1/CDC6/CDC45
KEGG_SPLICEOSOME	123	-0.658269234	-2.40118824	0.006410256	0.026353276	0.01754386	6126	tags=72%, list=25%, signal=55%	PLRG1/DDX39B/SNRPB2/PRPF3/SNRNP200/XAB2/LSM3/BCAS2/HNRNPC/RBM8A/NCBP1/HNRNPU/CRNKL1/PPIE/CTNNBL1/HNRNPA3/TRA2A/TCERG1/RBM17/DDX23/SART1/SRSF10/DDX46/SF3B5/PPIL1/SMNDC1/U2AF1/SF3B4/TRA2B/DHX15/THOC1/SF3A3/SNRNP70/PRPF6/THOC2/PRPF4/SRSF1/EIF4A3/LSM6/HNRNPM/ISY1/RBMX/PQBP1/SNRNP40/EFTUD2/DDX42/PPIH/SRSF7/PRPF40A/SRSF3/U2AF2/BUD31/NCBP2/SF3B2/MAGOH/PRPF19/SRSF2/SNRPA1/SRSF6/SRSF9/PRPF31/PUF60/CHERP/LSM7/SNRPC/SF3A2/SNRPG/WBP11/USP39/U2SURP/HNRNPA1/LSM2/PHF5A/SNRPB/SNRPD3/TXNL4A/ALYREF/SNRPA/LSM5/SNRPD1/SNRPE/HSPA1B/THOC3/PRPF40B/LSM4/SNRPD2/MAGOHB/SNRPF
KEGG_TYPE_I_DIABETES_MELLITUS	41	0.61816036	1.623641832	0.006747638	0.027137241	0.018065759	4410	tags=63%, list=18%, signal=52%	HLA-DQA2/CD28/HLA-E/CD86/LTA/HLA-DOB/PTPRN2/HLA-C/HLA-DMA/HLA-DMB/HLA-DOA/HLA-DPA1/HLA-G/HLA-B/HLA-F/HLA-A/HLA-DPB1/CPE/HLA-DRA/CD80/FASLG/HLA-DRB1/IL2/PRF1/IL12B/TNF

KEGG_ALZHEIMERS_DISEASE	145	-0.390345951	-1.474949332	0.007194245	0.028317771	0.01885166	5569	tags=53%, list=23%, signal=41%	NDUFB2/APH1A/PLCB3/NDUFV2/COX8A/CAPN1/IL1B/CALML6/IDE/NDUFS1/ATP2A2/NDUFC1/NDUFA3/SDHB/UQCRB/UQCRC1/NDUFB6/NDUFA2/COX4I1/NDUFS7/NDUFB10/NDUFB5/NDUFS3/NDUFA1/NDUFS2/MAPT/CALML3/NDUFA5/APP/UQCRC2/UQCR10/LPL/HSD17B10/PLCB1/COX7C/COX5A/NDUFA10/NDUFA6/PPP3R1/NDUFA8/CDK5/NDUFB3/RYR3/UQCR11/FADD/NDUFA4/FAS/GRIN2A/NDUFAB1/BID/NDUFS4/UQCRFS1/UQCRQ/COX7A2L/COX6C/NDUFS5/COX7A2/COX5B/NDUFB4/ITPR3/COX7B/NDUFB7/BAD/COX6B1/NDUFA7/COX6A1/NDUFS6/NDUFS8/NDUFV1/NDUFA9/CYCS/CYC1/UQCRH/NDUFB9/GAPDH/COX6B2
KEGG_FC_GAMMA_R_MEDIATED_PHAGOCYTOSIS	93	0.511510987	1.504936957	0.007434944	0.028655514	0.019076502	2858	tags=34%, list=12%, signal=31%	PLCG2/PTPRC/PRKCB/NCF1/WAS/PIK3CD/VAV1/FCGR2B/PIK3CG/LAT/LYN/DOCK2/PIK3R5/SCIN/INPP5D/FCGR2C/ARPC1B/PRKCD/RAC2/GAB2/MAPK3/FCGR1A/VASP/PIK3R1/FCGR3A/MARCKS/VAV3/SPHK1/WASF3/DNM1/PRKCA/GSN
KEGG_OTHER_GLYCAN_DEGRADATION	16	0.74202667	1.601876183	0.008169935	0.030327869	0.020189819	5266	tags=62%, list=21%, signal=49%	FUCA1/FUCA2/GBA/HEXA/NEU1/MAN2B2/HEXB/GLB1/MAN2B1/ENGASE
KEGG_HUNTINGTONS_DISEASE	160	-0.459299355	-1.76893552	0.008196721	0.030327869	0.020189819	4847	tags=52%, list=20%, signal=42%	NDUFC1/POLR2L/TAF4/NDUFA3/SDHB/UQCRB/UQCRC1/NDUFB6/HDAC1/NDUFA2/COX4I1/NDUFS7/NDUFB10/NDUFB5/NDUFS3/NDUFA1/NDUFS2/NDUFA5/AP2A1/SP1/UQCRC2/UQCR10/PPID/VDAC3/POLR2J2/POLR2E/PLCB1/COX7C/COX5A/SLC25A4/NDUFA10/NDUFA6/DCTN2/POLR2J/AP2S1/TBP/BBC3/POLR2K/NDUFA8/TFAM/NDUFB3/TBPL1/UQCR11/SLC25A5/TAF4B/NDUFA4/DNAL4/NDUFAB1/CLTB/NDUFS4/BDNF/UQCRFS1/UQCRQ/COX7A2L/COX6C/TP53/SOD1/NDUFS5/COX7A2/POLR2D/COX5B/POLR2F/NDUFB4/HDAC2/COX7B/NDUFB7/COX6B1/VDAC2/NDUFA7/COX6A1/SLC25A6/NDUFS6/VDAC1/NDUFS8/NDUFV1/POLR2I/NDUFA9/POLR2H/CYCS/CYC1/UQCRH/NDUFB9/COX6B2
KEGG_TOLL_LIKE_RECEPTOR_SIGNALING_PATHWAY	101	0.498584879	1.478670271	0.008610086	0.031232665	0.020792158	5003	tags=39%, list=20%, signal=31%	CTSK/LY96/TLR7/TLR9/PIK3CD/CXCL9/TLR4/PIK3CG/TLR8/CD86/PIK3R5/CD14/FOS/IRF7/MAPK3/MYD88/MAP3K8/STAT1/IFNAR2/PIK3R1/CXCL11/CD80/TLR2/TICAM2/IFNAR1/CCL5/IRF5/MAP2K3/MAPK14/RIPK1/TLR1/TLR6/CASP8/IL12B/TNF/AKT1/IRAK4/TIRAP/TRAF3
KEGG_SMALL_CELL_LUNG_CANCER	84	-0.422265999	-1.482718258	0.00952381	0.033882784	0.022556391	4155	tags=42%, list=17%, signal=35%	PIAS2/PIAS4/COL4A6/RELA/RXRA/CCNE1/E2F2/ITGA2/CDK2/LAMC2/COL4A2/TP53/PIK3CB/CCNE2/TRAF2/LAMB1/ITGA6/PIK3R2/BCL2/TRAF4/LAMA3/LAMB3/NFKBIA/PTK2/CYCS/MYC/SKP2/CCND1/CKS1B/CDK4/E2F1/LAMA5/ITGAV/PTGS2
KEGG_FC_EPSILON_RI_SIGNALING_PATHWAY	79	0.524996285	1.524102046	0.010050251	0.035081066	0.023354109	3760	tags=37%, list=15%, signal=31%	PLA2G2D/PLCG2/BTK/PRKCB/PIK3CD/VAV1/PIK3CG/LAT/FYN/LYN/PIK3R5/LCP2/PLA2G2A/INPP5D/PRKCD/RAC2/GAB2/MAPK3/FCER1G/MS4A2/PIK3R1/SOS2/VAV3/PRKCA/PLA2G10/MAP2K3/MAPK14/GRB2/SYK
KEGG_JAK_STAT_SIGNALING_PATHWAY	154	0.458437416	1.427214701	0.010344828	0.035440613	0.023593466	4550	tags=36%, list=19%, signal=29%	IL10RA/IL7R/PIK3CD/PTPN6/IRF9/PIK3CG/STAT4/PIK3R5/PIM1/IL21R/IL2RB/IL3RA/IL6R/IL12RB1/CSF2RA/STAT5B/CSF3R/LEPR/IL2RG/IFNLR1/IL5RA/CLCF1/IL9R/CCND3/STAT1/IL2RA/IFNAR2/CBL/CSF2RB/PIK3R1/JAK1/IL6ST/IL4R/SOS2/IFNAR1/IL21/IL10RB/CNTF/STAT5A/IL12RB2/CISH/GRB2/IL24/IL2/CBLB/JAK3/SPRY1/CTF1/IL12B/IL10/OSM/IL7/IL22RA2/SOCS3/AKT1
KEGG_CYSTEINE_AND_METHIONINE_METABOLISM	34	-0.536499986	-1.569000809	0.010600707	0.035656923	0.023737468	3958	tags=68%, list=16%, signal=57%	AHCYL1/MTR/GOT2/LDHC/AMD1/SRM/LDHB/DNMT3B/DNMT3L/MAT1A/APIP/ADI1/MAT2A/SMS/DNMT1/DNMT3A/ENOPH1/MPST/GOT1/CBS/LDHA/AHCY
KEGG_GLYCOSPHINGOLIPID_BIOSYNTHESIS_GANGLIO_SERIES	15	0.740204794	1.571910849	0.012965964	0.042833989	0.028515373	4252	tags=73%, list=17%, signal=61%	ST6GALNAC4/ST3GAL5/ST6GALNAC6/B3GALT4/ST3GAL1/ST6GALNAC5/HEXA/ST6GALNAC3/HEXB/GLB1/ST3GAL2
KEGG_PURINE_METABOLISM	151	-0.372369533	-1.424783249	0.014285714	0.046365915	0.03086664	3891	tags=37%, list=16%, signal=31%	NT5C/HPRT1/POLR3K/POLR2J2/POLR3D/ADCY3/POLE3/POLR2E/ENTPD6/POLR3A/PAPSS1/DGUOK/POLA2/POLR2J/CANT1/POLR1B/POLR2K/IMPDH2/AK2/PKM/POLR1C/POLR1D/PRIM2/ADSL/PDE9A/POLR3B/POLA1/POLE2/PNPT1/POLR3H/POLR1A/NME1-NME2/NME4/GART/ADK/PPAT/POLR2D/POLR2F/ADCY10/PRIM1/RRM1/GMPS/POLR2I/AK4/ATIC/POLR2H/POLE/PAICS/NT5M/RRM2/POLD2/NME2/POLD1/NME1/PAPSS2
KEGG_ECM_RECEPTOR_INTERACTION	83	0.508055175	1.475058125	0.015151515	0.048328109	0.032172909	2267	tags=33%, list=9%, signal=30%	COL6A3/COL3A1/COL1A2/ITGA4/THBS2/COL1A1/COL6A2/COL5A2/LAMA4/THBS1/COL6A1/TNC/ITGA1/TNXB/ITGB7/GP1BA/COMP/VWF/LAMA2/ITGA11/COL11A1/ITGA5/FN1/ITGA8/COL5A3/LAMB2/CD36

Gene set enrichment analysis (GSEA) for ZNF667-AS1 using GSE12452.

ID	setSize	enrichmentScore	NES	pvalue	p.adjust	qvalues	rank	leading_edge	core_enrichment
KEGG_PATHWAYS_IN_CANCER	319	-0.409967305	-1.656121601	0.001567398	0.013814217	0.008646167	3932	tags=27%, list=18%, signal=22%	HIF1A/RAC1/WNT10B/CRK/NFKBIA/CCDC6/STAT1/CDK6/PTCH1/CDKN1B/CRKL/SOS1/EP300/TPR/KRAS/BIRC2/LAMC2/SMAD2/PIK3CA/EGLN1/TP53/RBX1/WNT2/TFG/BRCA2/JUN/BIRC3/CDKN1A/MDM2/PIK3CB/PTEN/IGF1R/ITGB1/SMAD4/KITLG/HSP90B1/HDAC1/FAS/MAPK8/PTK2/VEGFA/RALA/BCL2/RAD51/FGF1/SKP2/GSTP1/FZD3/FH/CHUK/AKT3/E2F3/MSH6/HSP90AB1/ITGA6/LAMC1/CYCS/BIRC5/BID/FZD6/FADD/HDAC2/CUL2/FGF2/ITGAV/NRAS/LAMA1/CCNE2/MSH2/LAMB3/ARNT2/CKS1B/COL4A2/CDK4/PTGS2/CXCL8/BMP2/WNT5A/COL4A1/MYC/FZD7/FN1/KIT/LAMB1/MMP1
KEGG_FOCAL_ADHESION	196	-0.434579023	-1.674098484	0.00170068	0.013814217	0.008646167	3981	tags=28%, list=19%, signal=23%	ROCK2/RAC1/CRK/FLNA/KDR/ITGA1/CRKL/SOS1/BIRC2/DOCK1/LAMC2/PIK3CA/COL6A6/PPP1R12A/JUN/VCL/BIRC3/PDGFC/PIK3CB/PTEN/PAK3/IGF1R/ITGB8/ITGB1/RAP1B/MAPK8/PTK2/VEGFA/THBS4/BCL2/AKT3/CAV2/PPP1CC/ITGA6/LAMC1/CCND2/CAV1/ITGAV/COL11A1/COL5A1/COL1A1/THBS2/LAMA1/COL6A3/LAMB3/IBSP/COL4A2/COL1A2/COL4A1/COL3A1/FN1/SPP1/COL5A2/LAMB1
KEGG_PURINE_METABOLISM	149	-0.475619965	-1.794410551	0.001718213	0.013814217	0.008646167	3613	tags=35%, list=17%, signal=29%	PDE6D/POLR2G/PRPS1/POLR2F/DGUOK/PDE8A/POLR3C/POLR2I/POLR3G/PKM/RRM2B/POLD1/PRIM2/PNP/NT5M/DCK/POLR3F/POLR1D/PRPS2/NUDT9/AK4/POLR2B/POLE3/PFAS/GART/POLD2/NUDT5/ADK/AK2/POLR1B/NME4/NT5C3A/POLR2K/HPRT1/GMPS/POLR1C/POLA1/RRM1/POLE2/POLR2D/POLR2H/PRIM1/ADSL/ATIC/PAICS/PPAT/PNPT1/PAPSS2/RRM2/ADA/NME1
KEGG_CELL_CYCLE	123	-0.684992958	-2.509617213	0.00174216	0.013814217	0.008646167	3981	tags=62%, list=19%, signal=51%	YWHAH/YWHAZ/RBL1/SKP1/CDK6/MCM5/CDKN1B/EP300/CDK7/SMAD2/SMC1A/TFDP2/TP53/RBX1/CDKN1A/MDM2/STAG2/BUB1/YWHAE/SMAD4/TFDP1/CDC25B/ANAPC1/BUB3/CUL1/CCNB2/HDAC1/CDC27/CHEK1/ANAPC7/WEE1/RAD21/ANAPC10/CDC23/ANAPC5/YWHAQ/ESPL1/SKP2/ORC5/ORC4/ORC1/ORC6/E2F3/ATR/MCM4/CCND2/CDC25A/HDAC2/ORC2/YWHAG/CDC20/E2F5/ORC3/MCM6/CCNA2/GADD45A/PRKDC/CHEK2/CCNE2/MCM7/MCM3/PTTG1/MCM2/CDC7/CDC45/CDK1/PCNA/CDC6/CDK4/DBF4/MYC/MAD2L1/TTK/BUB1B/CCNB1
KEGG_UBIQUITIN_MEDIATED_PROTEOLYSIS	128	-0.512255002	-1.890791791	0.001760563	0.013814217	0.008646167	5723	tags=59%, list=27%, signal=43%	CDC16/UBE2E2/PML/PRPF19/ANAPC13/UBE2R2/UBE2E1/UBE2Q1/TRIM37/UBE2M/CUL4B/MID1/CBLB/PIAS4/UBE2D1/KLHL9/HERC4/FBXW11/UBE2G2/ANAPC11/PIAS1/PIAS2/KEAP1/UBE2I/UBE2B/SAE1/UBE3C/UBA3/CUL4A/SKP1/UBA6/RNF7/TRIP12/UBE2N/BIRC2/UBE3A/UBE2L6/UBR5/UBE2QL1/RBX1/UBE2K/SOCS1/UBE2Z/HERC2/BIRC3/UBE2W/FBXW7/MDM2/UBE2L3/DDB2/UBE2G1/UBE2D2/ANAPC1/RCHY1/CUL1/CDC27/ANAPC7/ANAPC10/CDC23/ANAPC5/WWP1/SKP2/UBE2F/UBA2/BRCA1/BIRC6/NEDD4L/NEDD4/CUL2/CDC20/UBE2E3/FANCL/KLHL13/UBE2C
KEGG_SPLICEOSOME	121	-0.706658359	-2.577274513	0.001763668	0.013814217	0.008646167	3873	tags=62%, list=18%, signal=51%	PRPF31/SNRNP27/DHX16/LSM4/DDX42/SF3B1/CDC40/LSM3/SF3A2/DDX5/EFTUD2/PPIH/PRPF38A/TRA2B/SNRNP40/PRPF4/SNRPB2/SNRPC/SF3B6/HNRNPC/DDX46/PRPF38B/TCERG1/TRA2A/SRSF1/SF3B4/PLRG1/AQR/USP39/SRSF6/NCBP1/THOC2/RBMX/HNRNPU/DDX23/EIF4A3/SRSF3/SRSF7/SMNDC1/PRPF40A/PPIL1/SNRPA/SNRPB/PHF5A/BCAS2/HNRNPA3/NCBP2/LSM7/SNRPA1/PRPF3/LSM6/LSM8/SF3A3/DHX15/SNRPD2/LSM2/SNRPD3/SRSF9/WBP11/MAGOH/TXNL4A/SNRPE/SRSF2/SNRPG/HNRNPA1/SRSF10/CRNKL1/THOC1/ALYREF/SNRPF/U2SURP/MAGOHB/SNRPD1/LSM5
KEGG_PYRIMIDINE_METABOLISM	95	-0.575030315	-2.016658973	0.001766784	0.013814217	0.008646167	3074	tags=44%, list=14%, signal=38%	UPP1/POLR3C/POLR2I/DTYMK/POLR3G/TYMP/RRM2B/POLD1/PRIM2/PNP/NT5M/DCK/CTPS2/POLR3F/POLR1D/POLR2B/UMPS/POLE3/POLD2/POLR1B/NME4/NT5C3A/POLR2K/POLR1C/DCTD/POLA1/RRM1/POLE2/POLR2D/CMPK2/UCK2/AK3/POLR2H/DUT/PRIM1/TK1/TYMS/PNPT1/RRM2/CTPS1/NME1

KEGG_PARKINSONS_DISEASE	98	-0.540984303	-1.905747412	0.001776199	0.013814217	0.008646167	4921	tags=56%, list=23%, signal=43%	PPID/SNCAIP/NDUFC2/UBE2G2/SDHC/NDUFS4/NDUFB3/SDHB/COX6A1/CASP3/NDUFB10/NDUFV2/NDUFA7/VDAC3/NDUFA1/SLC25A5/COX6B1/NDUFB6/NDUFV1/UQCRB/UBE2L6/UQCRQ/COX5B/GPR37/COX5A/NDUFB5/NDUFA6/COX7C/NDUFA5/NDUFB4/UBE2L3/COX7B/COX7A2L/NDUFA4/NDUFV3/NDUFS2/UBE2G1/COX7A2/NDUFS8/VDAC2/NDUFAB1/PARK7/NDUFS6/COX6C/UQCRC2/VDAC1/UQCRFS1/NDUFA8/NDUFS5/CYCS/UQCRH/CYC1/NDUFB9/NDUFA9
KEGG_RNA_DEGRADATION	53	-0.636311663	-2.057256188	0.001808318	0.013814217	0.008646167	4073	tags=58%, list=19%, signal=47%	PATL1/EXOSC4/LSM1/LSM4/CNOT6/PAPOLG/EXOSC2/LSM3/TTC37/ZCCHC7/C1D/DIS3/EXOSC3/EXOSC9/ENO2/WDR61/PARN/CNOT7/EXOSC5/ENO1/EXOSC8/HSPA9/LSM7/XRN2/LSM6/LSM8/LSM2/HSPD1/LSM5/PNPT1
KEGG_CYSTEINE_AND_METHIONINE_METABOLISM	33	-0.659471928	-1.956417798	0.001821494	0.013814217	0.008646167	4637	tags=55%, list=22%, signal=43%	LDHC/SDS/SRM/GOT1/DNMT3B/ADI1/IL4I1/MAT2A/APIP/DNMT1/AMD1/SMS/ENOPH1/MTR/AHCY/LDHA/LDHB
KEGG_AMINOACYL_TRNA_BIOSYNTHESIS	41	-0.687863796	-2.147688858	0.001834862	0.013814217	0.008646167	3338	tags=56%, list=16%, signal=47%	TARSL2/WARS2/EPRS/HARS2/FARSA/MTFMT/SARS/NARS/RARS/EARS2/YARS/MARS2/TARS/IARS2/LARS/FARSB/MARS/GARS/IARS/DARS/YARS2/NARS2
KEGG_PROTEASOME	41	-0.747410863	-2.33361022	0.001834862	0.013814217	0.008646167	3998	tags=78%, list=19%, signal=64%	IFNG/PSMD4/PSMD3/PSME1/PSMB6/PSMB1/PSME2/PSMB7/PSMA3/PSMB4/PSMD13/PSME4/PSMC3/PSMD8/PSME3/PSMC5/PSMD2/PSMD1/PSMD11/PSMB5/PSMC4/POMP/PSMA5/PSMC2/PSMA7/PSMA2/PSMB3/PSMD14/PSMA4/PSMD12/PSMA1
KEGG_P53_SIGNALING_PATHWAY	67	-0.594368599	-1.982317917	0.001841621	0.013814217	0.008646167	3410	tags=43%, list=16%, signal=36%	BBC3/TP53/SERPINE1/IGFBP3/CDKN1A/MDM2/PTEN/RRM2B/EI24/DDB2/RCHY1/CCNB2/FAS/CHEK1/SESN1/ATR/CYCS/CCND2/BID/CCNG1/GADD45A/CHEK2/CCNE2/CDK1/CDK4/RRM2/CCNB1/PMAIP1
KEGG_BASAL_TRANSCRIPTION_FACTORS	31	-0.644653418	-1.876642714	0.001851852	0.013814217	0.008646167	5158	tags=61%, list=24%, signal=47%	TAF11/TAF4/TAF7/TAF5L/TAF2/GTF2A2/TAF13/GTF2E1/TBP/GTF2H4/GTF2E2/STON1/GTF2H1/TBPL1/TAF5/GTF2H3/TAF4B/GTF2F2
KEGG_DNA_REPLICATION	36	-0.778539216	-2.348458481	0.001851852	0.013814217	0.008646167	2845	tags=72%, list=13%, signal=63%	RNASEH1/POLD1/PRIM2/RPA1/POLE3/POLD2/RPA3/RFC2/SSBP1/RPA4/MCM4/POLA1/POLE2/FEN1/RNASEH2A/RFC3/PRIM1/MCM6/MCM7/RFC5/MCM3/MCM2/PCNA/RFC4/DNA2
KEGG_SMALL_CELL_LUNG_CANCER	84	-0.563349751	-1.92866877	0.001858736	0.013814217	0.008646167	3336	tags=35%, list=16%, signal=29%	LAMC2/PIK3CA/TP53/BIRC3/PIK3CB/PTEN/ITGB1/PTK2/BCL2/SKP2/CHUK/AKT3/E2F3/ITGA6/LAMC1/CYCS/ITGAV/LAMA1/CCNE2/LAMB3/CKS1B/COL4A2/CDK4/PTGS2/COL4A1/MYC/FN1/LAMB1
KEGG_ECM_RECEPTOR_INTERACTION	82	-0.657383035	-2.247807435	0.001865672	0.013814217	0.008646167	879	tags=26%, list=4%, signal=25%	LAMC1/ITGAV/COL11A1/COL5A1/COL1A1/THBS2/LAMA1/COL6A3/LAMB3/IBSP/SDC2/COL4A2/COL1A2/HMMR/COL4A1/COL3A1/FN1/SPP1/COL5A2/LAMB1
KEGG_RIBOSOME	81	-0.52197189	-1.780655911	0.001869159	0.013814217	0.008646167	5840	tags=60%, list=27%, signal=44%	RPS10/RPL41/RPL23A/RPS15/RPS2/RPL14/RPL13A/UBA52/RPS4X/RPL10A/RPS8/RPS28/RPS3/FAU/RPL12/RPL19/RPS3A/RPL23/RPL30/RPS13/RPS19/RPS27A/RPS12/RPS23/RPS20/RPL35/RPL5/RPL24/RPL35A/RPS17/RPL7/RPL22/RPL4/RPL8/RPS5/RPL37A/RPS15A/RPLP0/RPL36/RPS7/RPS27L/RSL24D1/RPL31/RPS24/RPS21/RPL22L1/RPL26L1/MRPL13
KEGG_NUCLEOTIDE_EXCISION_REPAIR	43	-0.591845465	-1.853271775	0.001876173	0.013814217	0.008646167	4630	tags=60%, list=22%, signal=47%	POLE/CCNH/LIG1/CUL4A/GTF2H5/CDK7/RAD23B/RBX1/RFC1/POLD1/GTF2H4/DDB2/GTF2H1/RPA1/POLE3/POLD2/RPA3/RFC2/RPA4/POLE2/GTF2H3/RFC3/RFC5/PCNA/RFC4
KEGG_MISMATCH_REPAIR	22	-0.752037876	-2.029395919	0.001912046	0.013814217	0.008646167	2845	tags=68%, list=13%, signal=59%	POLD1/RPA1/POLD2/RPA3/RFC2/SSBP1/RPA4/MSH6/RFC3/RFC5/MSH2/EXO1/PCNA/RFC4
KEGG_HOMOLOGOUS_RECOMBINATION	26	-0.696557159	-1.959606827	0.001926782	0.013814217	0.008646167	2913	tags=58%, list=14%, signal=50%	POLD1/BLM/RPA1/RAD50/POLD2/RPA3/RAD51/EME1/SSBP1/RPA4/NBN/RAD54L/RAD51C/RAD54B
KEGG_ASTHMA	27	0.648171175	1.810920818	0.002074689	0.013814217	0.008646167	3450	tags=52%, list=16%, signal=44%	HLA-DMA/HLA-DMB/HLA-DRB1/TNF/HLA-DPB1/HLA-DOB/HLA-DQB1/FCER1A/HLA-DPA1/HLA-DRA/HLA-DOA/RNASE3/CD40LG/HLA-DRB4
KEGG_INTESTINAL_IMMUNE_NETWORK_FOR_IGA_PRODUCTION	44	0.645745927	2.006385502	0.002150538	0.013814217	0.008646167	2738	tags=50%, list=13%, signal=44%	PIGR/TNFRSF17/CXCL12/TNFRSF13C/HLA-DMA/HLA-DMB/AICDA/CCL28/HLA-DRB1/HLA-DPB1/ITGB7/HLA-DOB/HLA-DQB1/HLA-DPA1/ITGA4/HLA-DRA/TNFRSF13B/TNFSF13/IL2/HLA-DOA/CCL27/CD28
KEGG_HEMATOPOIETIC_CELL_LINEAGE	84	0.544230633	1.928947295	0.002155172	0.013814217	0.008646167	3279	tags=45%, list=15%, signal=38%	MS4A1/CD19/CD1C/CD1D/IL1R2/CD55/CR2/CR1/CD22/CD3D/IL7R/HLA-DRB1/TNF/IL6R/CD37/CD2/CD59/ITGA4/FCER2/CD8A/IL4R/GP1BA/HLA-DRA/CD3G/FLT3LG/TPO/CSF1R/IL7/IL1R1/CD1E/CD38/CSF2RA/CD8B/CD3E/IL5RA/CD5/CD7/FLT3

KEGG_B_CELL_RECEPTOR_SIGNALING_PATHWAY	74	0.583146783	2.009818718	0.002164502	0.013814217	0.008646167	3054	tags=45%, list=14%, signal=38%	CD19/CD72/CR2/BTK/PLCG2/FOS/PTPN6/CD79A/PRKCB/VAV1/LYN/CD22/CD79B/SYK/PIK3CD/CHP2/PPP3CA/CARD11/RAC2/BLNK/PIK3CG/INPP5D/RASGRP3/MAPK3/FCGR2B/PPP3CC/PIK3AP1/AKT1/CHP1/NFATC1/NFATC2/GRB2/NFAT5
KEGG_RETINOL_METABOLISM	48	0.580725057	1.832629649	0.002164502	0.013814217	0.008646167	2785	tags=27%, list=13%, signal=24%	DHRS9/ALDH1A1/ADH1C/ADH7/CYP2C18/ADH1B/UGT2B28/RDH10/RDH12/CYP2A7/ADH1A/CYP2C8/ADH6
KEGG_DRUG_METABOLISM_CYTOCHROME_P450	59	0.649869614	2.134587796	0.002192982	0.013814217	0.008646167	2785	tags=41%, list=13%, signal=35%	GSTA1/ALDH3A1/ADH1C/GSTA3/ADH7/FMO2/CYP2E1/ALDH1A3/FMO3/MAOA/ALDH3B1/CYP2C18/ADH1B/ALDH3B2/UGT2B28/GSTK1/FMO5/GSTM1/CYP2A7/ADH1A/GSTO2/CYP2C8/MAOB/ADH6
KEGG_METABOLISM_OF_XENOBIOTICS_BY_CYTOCHROME_P450	57	0.648976021	2.103887486	0.002207506	0.013814217	0.008646167	2785	tags=39%, list=13%, signal=34%	GSTA1/ALDH3A1/AKR1C3/ADH1C/GSTA3/ADH7/CYP2E1/CYP2F1/ALDH1A3/CYP1B1/ALDH3B1/CYP2C18/ADH1B/ALDH3B2/EPHX1/UGT2B28/GSTK1/GSTM1/ADH1A/GSTO2/CYP2C8/ADH6
KEGG_LEUKOCYTE_TRANSENDOTHELIAL_MIGRATION	113	0.498352227	1.855892058	0.002257336	0.013814217	0.008646167	3281	tags=35%, list=15%, signal=29%	CLDN10/RHOH/NCF1/ITGAL/PLCG2/CXCL12/CLDN3/CLDN23/ITGB2/PRKCB/VAV1/CLDN8/MMP9/CLDN7/EZR/SIPA1/ITK/CYBA/NCF4/PIK3CD/CLDN11/RASSF5/JAM2/RAC2/PIK3CG/ITGA4/GNAI2/CLDN4/MYL12B/JAM3/CLDN9/TXK/RHOA/CD99/GNAI1/MYLPF/CYBB/PECAM1/CLDN15
KEGG_CELL_ADHESION_MOLECULES_CAMS	126	0.454653345	1.716117136	0.002314815	0.013814217	0.008646167	3336	tags=40%, list=16%, signal=34%	CLDN10/SELL/ITGAL/PTPRC/ICAM3/CLDN3/HLA-DMA/CLDN23/HLA-DMB/ITGB2/CD22/CLDN8/CLDN7/HLA-E/HLA-DRB1/HLA-DPB1/ITGB7/HLA-DOB/HLA-DQB1/ALCAM/CLDN11/SELP/JAM2/CD2/HLA-DPA1/ITGA4/HLA-F/HLA-B/CD8A/HLA-DRA/HLA-G/ITGA8/CLDN4/SELPLG/GLG1/ITGA9/CDH3/JAM3/HLA-A/NRXN3/CLDN9/SDC1/HLA-DOA/CD28/HLA-C/CD99/CD8B/ICAM2/PECAM1/CLDN15/CD40LG
KEGG_NATURAL_KILLER_CELL_MEDIATED_CYTOTOXICITY	126	0.432408288	1.632151795	0.002314815	0.013814217	0.008646167	3190	tags=33%, list=15%, signal=28%	ITGAL/PLCG2/PTPN6/ITGB2/PRKCB/VAV1/LCK/CD48/HLA-E/SYK/LAT/TNF/FYN/ZAP70/PIK3CD/CHP2/PPP3CA/RAC2/PIK3CG/HCST/CD247/NCR3/HLA-B/SH2D1B/MAPK3/PPP3CC/HLA-G/SH2D1A/TNFRSF10D/IFNGR1/RAET1E/CHP1/HLA-A/LCP2/NFATC1/HLA-C/NFATC2/GRB2/NFAT5/ICAM2/IFNAR1
KEGG_GLYCOSYLPHOSPHATIDYLINOSITOL_GPI_ANCHOR_BIOSYNTHESIS	24	−0.635045047	−1.752957552	0.003816794	0.02143809	0.013417866	4450	tags=50%, list=21%, signal=40%	PIGP/PIGB/PIGK/PIGC/PIGU/PIGN/PIGX/PIGF/PIGW/PIGA/PGAP1
KEGG_PROTEIN_EXPORT	22	−0.683203077	−1.843643227	0.003824092	0.02143809	0.013417866	5643	tags=82%, list=26%, signal=60%	SRP68/SPCS3/OXA1L/SEC61B/SRP54/SEC61A1/SPCS2/IMMP1L/HSPA5/SEC63/SRP19/SEC61A2/SRP72/SEC11A/IMMP2L/SRP9/SEC61G
KEGG_LEISHMANIA_INFECTION	68	0.483172352	1.639465015	0.004310345	0.023453347	0.014679193	1741	tags=29%, list=8%, signal=27%	C3/NCF1/FOS/PTPN6/HLA-DMA/HLA-DMB/ITGB2/CR1/PRKCB/HLA-DRB1/TNF/HLA-DPB1/CYBA/HLA-DOB/HLA-DQB1/NCF4/HLA-DPA1/ITGA4/MAPK3/HLA-DRA
KEGG_T_CELL_RECEPTOR_SIGNALING_PATHWAY	107	0.421656562	1.542489909	0.004716981	0.024932615	0.01560505	3580	tags=38%, list=17%, signal=32%	PTPRC/FOS/PTPN6/VAV1/LCK/CD3D/LAT/TNF/FYN/ITK/ZAP70/PIK3CD/CHP2/MAP3K8/PPP3CA/CARD11/PIK3CG/CD247/CD8A/RASGRP1/MAPK3/CD3G/PPP3CC/AKT1/CHP1/LCP2/IL2/NFATC1/RHOA/CBL/PDPK1/CD28/NFATC2/CD8B/GRB2/CD3E/NFAT5/PAK6/CD40LG/PRKCQ/MAP3K14
KEGG_HUNTINGTONS_DISEASE	158	−0.426029245	−1.626321532	0.005025126	0.025823562	0.016162685	5110	tags=51%, list=24%, signal=39%	CLTC/TAF4/SP1/NDUFS1/PPID/HIP1/NDUFC2/SDHC/NDUFS4/NDUFB3/SDHB/CREB1/COX6A1/CASP3/BAX/NDUFB10/NDUFV2/AP2S1/NDUFA7/VDAC3/NDUFA1/SLC25A5/COX6B1/NDUFB6/POLR2G/NDUFV1/EP300/BBC3/POLR2F/UQCRB/UQCRQ/COX5B/COX5A/PLCB1/NDUFB5/DCTN4/TP53/BDNF/POLR2I/NDUFA6/COX7C/NDUFA5/NDUFB4/SOD2/COX7B/COX7A2L/SOD1/NDUFA4/TBP/NDUFV3/NDUFS2/COX7A2/NDUFS8/VDAC2/NDUFAB1/NDUFS6/HDAC1/COX6C/UQCRC2/POLR2B/TBPL1/AP2B1/CREB5/VDAC1/UQCRFS1/NDUFA8/TFAM/NDUFS5/POLR2K/CYCS/HDAC2/PLCB4/UQCRH/POLR2D/TAF4B/POLR2H/CYC1/NDUFB9/NDUFA9

KEGG_ALZHEIMERS_DISEASE	143	-0.406785935	-1.524797272	0.005190311	0.025951557	0.016242795	4735	tags=44%, list=22%, signal=34%	SDHC/NDUFS4/NDUFB3/PSEN2/SDHB/ATP2A2/COX6A1/CASP3/ADAM17/NDUFB10/NDUFV2/GRIN2A/GSK3B/NDUFA7/NDUFA1/COX6B1/NDUFB6/RYR3/NDUFV1/ATF6/UQCRB/UQCRQ/COX5B/COX5A/PLCB1/CDK5/NDUFB5/NDUFA6/COX7C/NDUFA5/NDUFB4/APP/COX7B/COX7A2L/IL1B/NDUFA4/NDUFV3/NDUFS2/COX7A2/PPP3R1/NDUFS8/NDUFAB1/TNFRSF1A/NDUFS6/FAS/COX6C/UQCRC2/HSD17B10/NAE1/UQCRFS1/NDUFA8/NDUFS5/CYCS/BID/FADD/PLCB4/UQCRH/CYC1/NDUFB9/GAPDH/NDUFA9/LPL
KEGG_BLADDER_CANCER	41	-0.560088753	-1.748742094	0.005504587	0.026798648	0.01677298	3363	tags=29%, list=16%,	TP53/TYMP/CDKN1A/MDM2/VEGFA/E2F3/NRAS/CDK4/CXCL8/MYC/MMP1
KEGG_STEROID_HORMONE_BIOSYNTHESIS	43	0.537092687	1.65891046	0.006396588	0.028479064	0.017824734	1528	tags=23%, list=7%, signal=22%	AKR1C3/SRD5A2/HSD17B2/CYP1B1/SULT1E1/SRD5A3/STS/SULT2B1/UGT2B28/HSD17B8
KEGG_PRIMARY_IMMUNODEFICIENCY	35	0.561784346	1.649877206	0.006437768	0.028479064	0.017824734	1970	tags=40%, list=9%, signal=36%	CD19/BTK/PTPRC/TNFRSF13C/CD79A/LCK/AICDA/CD3D/IL7R/ZAP70/IL2RG/BLNK/CD8A/TNFRSF13B
KEGG_ALLOGRAFT_REJECTION	34	0.587822588	1.723509041	0.006465517	0.028479064	0.017824734	3774	tags=62%, list=18%, signal=51%	HLA-DMA/HLA-DMB/HLA-E/HLA-DRB1/TNF/HLA-DPB1/HLA-DOB/HLA-DQB1/HLA-DPA1/HLA-F/HLA-B/HLA-DRA/HLA-G/HLA-A/IL2/HLA-DOA/CD28/HLA-C/CD40LG/HLA-DRB4/IL12B
KEGG_GRAFT_VERSUS_HOST_DISEASE	34	0.580452918	1.701901001	0.006465517	0.028479064	0.017824734	2752	tags=53%, list=13%, signal=46%	HLA-DMA/HLA-DMB/HLA-E/HLA-DRB1/TNF/HLA-DPB1/HLA-DOB/HLA-DQB1/HLA-DPA1/HLA-F/HLA-B/HLA-DRA/HLA-G/HLA-A/IL2/HLA-DOA/CD28/HLA-C
KEGG_AUTOIMMUNE_THYROID_DISEASE	49	0.52094842	1.646002594	0.008695652	0.036963037	0.02313476	3450	tags=41%, list=16%, signal=34%	HLA-DMA/HLA-DMB/HLA-E/HLA-DRB1/HLA-DPB1/HLA-DOB/HLA-DQB1/HLA-DPA1/HLA-F/HLA-B/HLA-DRA/HLA-G/TPO/HLA-A/IL2/HLA-DOA/CD28/HLA-C/CD40LG/HLA-DRB4
KEGG_FC_EPSILON_RI_SIGNALING_PATHWAY	75	0.45366536	1.566656921	0.008791209	0.036963037	0.02313476	1714	tags=25%, list=8%, signal=23%	BTK/PLCG2/PRKCB/PRKCD/VAV1/LYN/SYK/LAT/PLA2G10/TNF/FYN/PLA2G2D/PIK3CD/FCER1A/RAC2/PIK3CG/MAP2K3/INPP5D/MAPK3
KEGG_RNA_POLYMERASE	29	-0.614176806	-1.768839316	0.009416196	0.037785948	0.023649811	3613	tags=48%, list=17%, signal=40%	POLR2G/POLR2F/POLR3C/POLR2I/POLR3G/POLR3F/POLR1D/POLR2B/POLR1B/POLR2K/POLR1C/POLR2D/POLR2H
KEGG_SYSTEMIC_LUPUS_ERYTHEMATOSUS	105	0.407675295	1.486932916	0.009456265	0.037785948	0.023649811	2738	tags=28%, list=13%, signal=24%	C3/HIST2H2BE/HLA-DMA/HLA-DMB/C7/H2AFJ/HLA-DRB1/TNF/HLA-DPB1/HLA-DOB/HLA-DQB1/C6/HIST1H2AE/HIST1H2BD/HLA-DPA1/HIST1H2BG/HIST1H2AC/HLA-DRA/FCGR2B/HIST1H2BC/C1R/H3F3A/HIST1H2AJ/C1S/HIST1H2AI/HLA-DOA/CTSG/C1QA/CD28
KEGG_GLYOXYLATE_AND_DICARBOXYLATE_METABOLISM	16	-0.675203727	-1.690927482	0.009803922	0.037785948	0.023649811	4553	tags=56%, list=21%, signal=44%	PGP/MTHFD2L/MDH2/MTHFD1L/GRHPR/MDH1/CS/MTHFD2
KEGG_ONE_CARBON_POOL_BY_FOLATE	16	-0.679506372	-1.701702688	0.009803922	0.037785948	0.023649811	2371	tags=56%, list=11%, signal=50%	MTFMT/GART/MTHFD2/DHFR/MTR/ATIC/TYMS/SHMT2
KEGG_BASE_EXCISION_REPAIR	33	-0.592109261	-1.756576809	0.010928962	0.040838852	0.02556059	4845	tags=58%, list=23%, signal=45%	POLD3/POLE/HMGB1/LIG1/SMUG1/PARP2/POLD1/PARP1/POLE3/POLD2/APEX1/TDG/NEIL3/POLE2/FEN1/NTHL1/UNG/PCNA
KEGG_ARACHIDONIC_ACID_METABOLISM	52	0.489231285	1.547409276	0.011037528	0.040838852	0.02556059	2944	tags=33%, list=14%, signal=28%	AKR1C3/PTGDS/GGT6/ALOX15/CYP2E1/GPX3/PLA2G10/GPX1/CYP2C18/PLA2G2D/CBR1/TBXAS1/CYP2U1/CYP2C8/HPGDS/ALOX5/ALOX12
KEGG_OXIDATIVE_PHOSPHORYLATION	100	-0.440650783	-1.566848635	0.012259194	0.044469627	0.027833052	5112	tags=55%, list=24%, signal=42%	NDUFB2/ATP6V1E1/COX17/NDUFS1/NDUFA11/ATP6V1B2/NDUFC2/SDHC/NDUFS4/NDUFB3/SDHB/COX6A1/NDUFB10/ATP6V1E2/NDUFV2/PPA2/NDUFA7/NDUFA1/COX6B1/NDUFB6/ATP6V1C2/COX15/NDUFV1/COX10/UQCRB/UQCRQ/COX5B/COX5A/NDUFB5/NDUFA6/COX7C/NDUFA5/NDUFB4/COX7B/COX7A2L/NDUFA4/NDUFV3/NDUFS2/COX7A2/NDUFS8/NDUFAB1/ATP6V1C1/NDUFS6/COX6C/UQCRC2/UQCRFS1/NDUFA8/NDUFS5/COX11/UQCRH/CYC1/PPA1/NDUFB9/NDUFA9
KEGG_CALCIUM_SIGNALING_PATHWAY	174	0.35574996	1.406727564	0.012594458	0.044807208	0.028044341	4147	tags=26%, list=19%, signal=21%	P2RX5/CALML5/OXTR/PLCG2/PRKCB/CALML3/CYSLTR1/GNAL/CHP2/GNA14/PPP3CA/ADRB1/ADCY7/ITPR1/ATP2A3/PLCE1/PRKACB/PPP3CC/HTR5A/PLCB2/TNNC1/EDNRB/TRPC1/CHP1/CACNA1F/P2RX1/CD38/CAMK2D/TNNC2/CACNA1H/ITPR2/HRH1/ADRA1B/GNAQ/CHRM1/MYLK2/GNAS/HTR2B/ERBB2/CACNA1A/CAMK2A/ADCY2/CHRM5/P2RX7/PTK2B
KEGG_VIRAL_MYOCARDITIS	67	0.468421662	1.579566323	0.013071895	0.045628314	0.028558262	3510	tags=39%, list=16%, signal=33%	CD55/ITGAL/HLA-DMA/HLA-DMB/ITGB2/HLA-E/HLA-DRB1/FYN/HLA-DPB1/HLA-DOB/HLA-DQB1/RAC2/HLA-DPA1/HLA-F/HLA-B/HLA-DRA/HLA-G/MYH14/HLA-A/HLA-DOA/CD28/HLA-C/CD40LG/SGCA/HLA-DRB4/MYH13

KEGG_VALINE_LEUCINE_AND_ ISOLEUCINE_BIOSYNTHESIS	11	-0.726070133	-1.654210401	0.014256619	0.048842121	0.030569749	2022	tags=45%, list=9%, signal=41%	PDHA1/IARS2/LARS/IARS
